# Sulfate and Dissolved Organic Carbon Concentrations Drive Distinct Microbial Community Patterns in Prairie Wetland Ponds

**DOI:** 10.1111/1758-2229.70069

**Published:** 2025-01-27

**Authors:** Zohra Zahir, Faraz Khan, Britt D. Hall

**Affiliations:** ^1^ Department of Biology University of Regina Regina Saskatchewan Canada

**Keywords:** biogeochemistry, climate change, microbial ecology

## Abstract

Prairie wetland ponds on the Great Plains of North America offer a diverse array of geochemical scenarios that can be informative about their impact on microbial communities. These ecosystems offer invaluable ecological services while experiencing significant stressors, primarily through drainage and climate change. In this first study systematically combining environmental conditions with microbial community composition to identify various niches in prairie wetland ponds, sediments had higher microbial abundance but lower phylogenetic diversity in ponds with lower concentrations of dissolved organic carbon ([DOC]; 10–18 mg/L) and sulfate ([SO_4_^2−^]; 37–58 mg/L) in water. As [DOC] and [SO_4_^2−^] increased, there was an initial decline in abundance but not phylogenetic diversity. Maximum values of both abundance and phylogenetic diversity occurred between 56 and 115 mg/L [DOC] and 5,000–6,000 mg/L [SO_4_^2−^] and decreased thereafter in ponds with 150–180 mg/L and 8,000–14,000 mg/L [DOC] and [SO_4_^2−^], respectively. These findings confirm that environmental variables shape the microbial communities and that key microbial taxa involved in sulfur and carbon cycling dominated these ponds potentially impacting vital biogeochemical processes such as bioavailability of heavy metals, carbon sequestration, and methane emissions.

## Graphical abstract

Microbial community composition in prairie wetland ponds is shaped by varying concentrations of sulfate (SO_4_^2−^) and dissolved organic carbon (DOC) resulting in shifts in richness/evenness and phylogenetic diversity. Our study emphasises the importance of understanding microbial responses to geochemical scenarios in freshwater ecosystems.

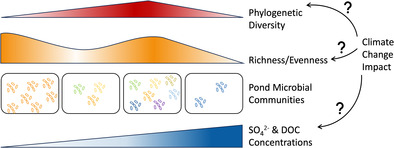

## 1 Introduction

Microbial communities play a pivotal role in the functioning and productivity of shallow wetland ponds (De Mandal et al. 2020; Sims et al. 2013). They serve as key drivers of biogeochemical processes, including nutrient cycling, organic matter decomposition, and the transformation of heavy metals and pollutants. For example, sulfur and carbon biogeochemical cycles are highly regulated by microbial communities (Pester et al. 2012). In carbon cycling, microbial communities are instrumental in regulating greenhouse gas emissions, carbon fixation, and organic carbon degradation, thereby influencing wetland ecosystem carbon balances (De Mandal et al. 2020; Kayranli et al. 2010). Microbial processes in sulfur cycling, including sulfate reduction and sulfur oxidation, shape the dynamics of sulfur transformations within these ecosystems (Pester et al. 2012). These processes are vital in wetland functions as they can influence the groundwater recharge by controlling the sulfur and carbon species and impacting the water quality and carbon sequestration (Bansal et al. 2023; Kurek et al. 2024). Wetlands also regulate other important biogeochemical cycles, such as nitrogen (N) and phosphorus (P) cycling, both of which control nutrient availability and eutrophication leading to harmful algal blooms (Jarvie et al. 2020). Wetlands can also act as hotspots for methylmercury production, a toxic compound that accumulates in the food chain and poses health risks to both wildlife and humans (Ackerman and Eagles‐Smith 2010; Branfireun et al. 1999; Branfireun, Heyes, and Roulet 1996; Hoggarth, Hall, and Mitchell 2015). As such, identifying the environmental factors that influence the composition of microbial communities that regulate many biogeochemical cycles is key to understanding and managing wetland ecosystems. While numerous studies have explored deterministic and stochastic factors affecting microbial communities across diverse ecosystems (e.g., in agricultural watersheds by Chen et al. 2018; in agricultural reservoirs and restored wetlands by Hartman et al. 2008; in salt march ponds by Kearns et al. 2017; in constructed wetlands by Verduzo Garibay et al. 2022; in coastal wetlands by Yang et al. 2021; in freshwater lakes by Zhao et al. 2022), research on microbial community dynamics in natural freshwater wetlands remains relatively limited. Notably, the microbial communities in wetlands within the Prairie Pothole Region (PPR) of the Great Plains of North America represent one such understudied area. This ecologically significant area presents a unique opportunity to explore correlations between microbes and their environment, including the distinct environmental niches created by the diverse geochemistry of wetland ponds.

Wetlands are important for sustainable prairie ecosystems, offering essential ecological services such as water purification, flood prevention, nutrient cycling, and providing habitats for diverse wildlife (Hartig 2005; Sando et al. 2007). The PPR covers a vast area of approximately 850,000 km^2^, spanning the Canadian provinces of Alberta, Saskatchewan, Manitoba, and five USA states (Janke, Anteau, and Stafford 2017). Within this expansive region, millions of small productive shallow (i.e., depth of less than two meters) wetland ponds of varying sizes (typically between 0.1 and 0.25 ha; Watmough and Schmoll 2007) are found (Hartig 2005; Johnson et al. 2010; Smith, Stoudt, and Gollop 1964). Most of the wetland ponds are temporary, typically retaining water for just 1 to 2 months, while some are seasonal, lasting around 2 to 3 months (Hall et al. 2023; Johnson et al. 2010). Despite the ephemeral nature of the ponds, these wetland systems support a remarkable diversity of wildlife, including threatened and endangered species (Knutsen and Euliss 2001) which underscores their critical role in maintaining ecological balance and preserving biodiversity.

Heterogeneity is a defining feature of PPR wetlands, which exhibit substantial variability in various characteristics across different locations and within each site, including heterogeneous geochemistry (Hoggarth, Hall, and Mitchell 2015), vegetation (Hargiss et al. 2023), bird distribution (Elliott, Igl, and Johnson 2020), hydrology, and permanency (Hall et al. 2023; Hayashi, van der Kamp, and Rosenberry 2016). One of the main characteristics of wetlands is that they are highly fertile and productive, which can be attributed to the accumulation of organic matter (OM) in saturated soil and sediment (Euliss et al. 2006; Seelig and DeKeyser 2006). The accumulation of OM in these wetlands is primarily a result of decreased OM decomposition and subsequent accumulation due to anoxic conditions in both water and soil (Reddy, DeLaune, and Inglett 2022). Within wetland ponds, concentrations of dissolved OM, mainly in the form of dissolved organic carbon (DOC), can be high (Arts et al. 2000; Waiser 2006) and can act as electron donors, energy, and carbon sources for microbial communities, impacting their activities (e.g., in agricultual, forested, and wetland streams by Fasching et al. 2020). High concentrations of OM can lead to an increase in the availability of P and N which can stimulate plant growth and alter the stoichiometry of the system (Jarvie et al. 2020). Conversely, changes in nutrient stoichiometry can also impact the quantity and quality of OM in wetland ecosystems. For example, increasing N to P ratios  with corresponding decreases in P, can lead to decreasing OM decomposition rates (Jarvie et al. 2020) allowing OM accumulation in wetland soil and sediment. This can have implications for carbon sequestration and greenhouse gas emissions (Zhang et al. 2017).

In addition to the OM buildup, wetland ponds can have high concentrations of various sulfur species, specifically sulfate (SO_4_^2−^), in water and sediments (Dalcin Martins et al. 2017; Zeng, Arnold, and Toner 2013). The primary source of SO_4_^2−^ is from underlying geological deposits (Goldhaber et al. 2016; Nachshon et al. 2013). Sulfur cycling is particularly important as it regulates bioavailability of heavy metals including copper, cadmium, nickel, zinc, and mercury (Wu et al. 2021). However, while elevated concentrations of SO_4_^2−^ ([SO_4_^2−^]) and DOC ([DOC]) are characteristic of many PPR wetlands, this is not universal, and some wetlands exhibit lower concentrations of both (Hoggarth, Hall, and Mitchell 2015) adding to the overall heterogeneity of these wetlands. Neither the impact of environmental heterogeneity, especially concentrations of DOC and SO_4_^2−^, on microbial community composition (richness/evenness/phylogenetic diversity) nor the potential influence of these microbial communities on other biogeochemical cycles have been fully explored in these wetland ponds.

To address this knowledge gap, we investigated the interactions between the geochemistry and their microbial communities in wetland ponds in central Saskatchewan, Canada. Specifically, we identified these microbial communities via 16S rRNA gene sequencing and used statistical models to examine relationships between identified microbial species and surrounding environmental conditions. We suggest that combining the richness and phylogenetic diversity provides a more comprehensive picture of microbial communities' composition than assessing richness alone as proposed by O'Dwyer, Kembel, and Green (2012). The insights from this study contribute to our knowledge of wetland ecology, biodiversity, and biogeochemical processes, and provide valuable information for the conservation and sustainable management of the Prairie Pothole wetlands.

## 2 Materials and Methods

### 2.1 Study Site

**FIGURE 1:**
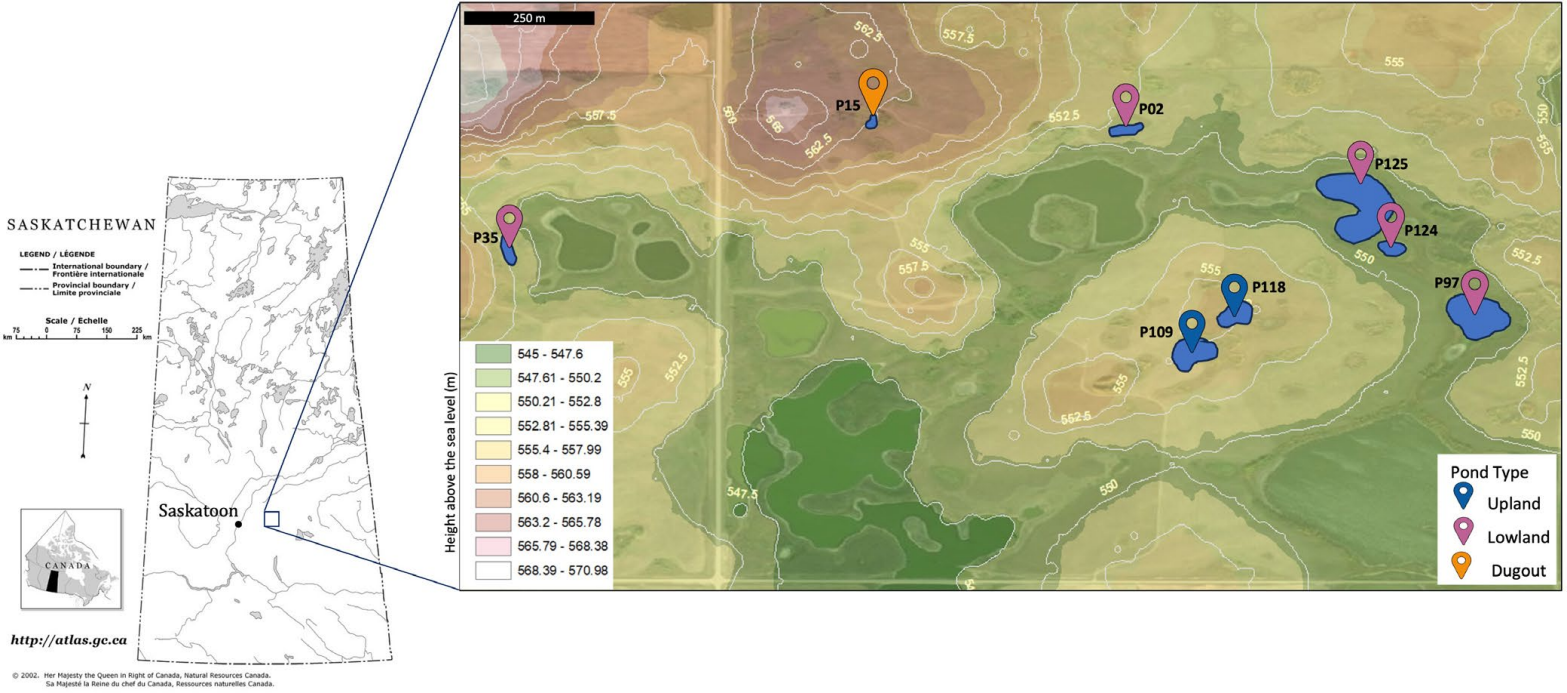
Left: Saskatchewan map. Right: Locations of the ponds that were sampled for geochemistry and microbial community analysis from St. Denis National Wildlife Area (SDNWA; Google n.d.; Government of Canada 2017). Each pin represents a pond in two different ecosites based on gradients of slopes and the mechanism of groundwater and runoff recharge and discharge in this area described by SDNWA management plan (Government of Canada 2017). Blue pins = Upland (P109, P118); Pink pins = Lowland (P02, P35, P97, P124, P125); and Orange pin = Dugout (P15; Upland). The coordinates, size, depth, and permanency for each pond can be found in Table S1. The blue lines represent the rough estimate of the ponds' sizes using Google Earth measurement tool. The contouring lines and colours show the elevation above the sea level calculated using ArcGIS and ArcMap software by Esri.

Samples were collected every 2 weeks from May to September 2021 from eight prairie wetlands located in the St. Denis National Wildlife Area (SDNWA) ~40 km east of Saskatoon, Saskatchewan (106.08° W, 52.20° N; Figure 1). Annual cultivated crops or wild and tame grasses and willow rings surround wetland ponds at the SDNWA (Pennock et al. 2014; Pennock et al. 2010). Annual precipitation at the site based on local observations from precipitation gauges and snow surveys (Bam et al. 2020) for the period 1994–2017 is approximately 360 mm/year, with around 80 mm/year falling as snow. The locally measured monthly mean air temperature for the same period varies between −14.7°C in January and 18.7°C in July (Bam et al. 2020). The SDNWA has two main ecosites (upland and lowland) based on slope gradients and the groundwater and runoff recharge and discharge mechanism (Government of Canada 2017). In this research, two ponds (P109, P118; P notation refers to ponds) are located at higher elevations (555–565 m) referred hereafter to as the ‘upland ecosite’, and five ponds (P02, P35, P97, P124, P125) are at lower elevations (545–552 m) referred hereafter as the ‘lowland ecosite’. In addition, P15 (also located in the upland ecosite) is a historical agricultural reservoir co‐locally known as a ‘dugout’. This reservoir has remained unused since 1967. Additional site characteristics are available in Hall et al. (2023).

### 2.2 Sample Collection and Measurements of Geochemical Parameters

For each pond, samples were taken along a transect running from ~5 cm water depth (i.e., flooded sites near the edge of the pond) and 0 m, 2 m, and 4 m inland from the water's edge, a transect chosen because 1, these ponds are shallow (5 to 70 cm; Table S1), homogenous, and constantly being mixed by wind and 2. in a related study, the microbial community of the sediment from the edge of the pond was no different than samples from the deepest section of the pond (Figure S1; Zahir et al., unpublished data). Sediment samples from immersed sites were obtained using acrylic core tubes 5 cm in diameter. Samples outside the water were obtained using a tulip bulb planter. Soil samples acquired immediately inland from the water's edge (0 m) were saturated, while those from the 2 m and 4 m soils were not. The top 2 cm of material collected was placed in a Ziploc bag and immediately stored on ice until arriving at the lab, where they were frozen at −20°C for subsequent DNA extraction and geochemical analysis. At each sampling site, we collected three replicates.

Water for the analysis of [SO_4_^2−^] and [DOC] and chlorophyll a (Chl.a) extraction was collected in a pre‐washed bottle rinsed with pond water, kept on ice, and within 24 h, filtered through Whatman GF/C 1.2 μm pore size filters and subsequently through HA Membrane 0.45 μm pore size filters (Millipore, USA), and stored at 4°C until analysis. DOC and SO_4_^2−^ concentrations were analysed by standard methods (Stainton, Capel, and Armstrong 1977) on an Aurora 1030D TOC Analyser (University of Regina) and a SmartChem 170 Discrete Analyser (University of Saskatchewan), respectively. Whatman filters were wrapped in aluminium foil and stored in the dark at −20°C for Chl.a extraction and analysis using an Agilent 8453 UV–visible spectrophotometer (University of Regina; Jeffrey and Humphrey 1975). Other water parameters (pH, conductivity, temperature (Temp), and oxidation–reduction potential (ORP)) were coincidentally collected from the surface water during the water sampling using a YSI ProQuatro multiparameter meter (Yellow Springs, USA).

### 2.3 DNA Extraction, Amplicon Library Preparation, and Sequencing

DNA was extracted from 0.25 g of sediment using a DNeasy PowerSoil Pro Kit (Qiagen, Germany). All DNA samples were quantified by the Thermo Scientific Qubit 2.0 Fluorometer dsDNA broad‐range assay kit. To assess DNA purity, the ratio of absorbance at 260 and 280 nm was measured using Thermo Scientific NanoDrop spectrophotometers. The V4 region of the 16S rRNA gene was amplified using a modified version of dual‐index primer pair 515F/806R as described in Kozich et al. 2013. Sequencing adaptors and barcodes were bound to ~10 ng of amplicon DNA using Nextera DNA Prep (Illumina Inc., San Diego, CA, USA). Ninety libraries were sequenced using Illumina MiSeq V2, 2 × 250bp pair‐end reads to ensure coverage of the whole region of V4 (254 bp) with 246 bp overlap. Library preparation and sequencing were performed by the UBC Sequencing and Bioinformatics Consortium (Vancouver, CAN). The raw sequence reads were deposited in the United States National Center for Biotechnology Information (NCBI) GenBank Short Read Archive (SRA) under accession number PRJNA1105329.

### 2.4 Bioinformatic and Statistical Analysis

After obtaining raw sequence reads from each sample, bioinformatic and statistical analyses were conducted to characterise the microbial communities and investigate their potential functions. First, the forward and reverse reads were merged, denoised, and chimera‐filtered using the *dada2* pipeline (v1.26.0; Callahan et al. 2016) within the R framework (R Core Team 2022). This process generated amplicon sequence variants (ASVs) representing the exact sequences that could be assigned to taxonomies (Callahan et al. 2016). The SILVA V138 database was employed to compare the ASVs and assign taxonomic identities (Callahan et al. 2016; Quast et al. 2013; Yilmaz et al. 2014). A total of 31,172 ASVs distributed across seven taxonomic ranks were identified by analysis of 16S rRNA gene sequencing data using the *dada2* pipeline and SILVA V138 database. In addition to these ASVs, 1652 ASVs could not be confidently assigned to any taxonomic group. Less prevalent ASVs (i.e., those with an abundance of less than 0.01, as well as chloroplast and mitochondrial sequences) were filtered out, and the sequenced results were assessed using the *decontam* package (Davis et al. 2018) to identify and remove potential contamination. These processes reduced the dataset from 31,172 to 25,274 ASVs (81%). To identify top taxa, a version of *ggnested* (a wrapper function around *ggplot2*) that allows for visualisation of data by setting nested taxa to 2, was performed using the *fantaxtic* package (Teunisse 2022). Sampling Depth and Good's Coverage (the fraction of ASVs that appeared more than once and their coverage, that is if the sequence depth was enough; Good 1953) were calculated using 100 * (1‐(n_sings/n_seqs)), where n_sings is the number of singletons and n_seqs is the total number of sequences (Table S2; Schloss 2019). Samples were then normalised using the *rarefy‐even‐depth*() function from the *phyloseq* package (Figure S2A; v1.44.0; McMurdie and Holmes 2013) with the sample size (i.e., rarefying depth) of 57,608 calculated using ‘min rowSums(otu_table)’ with 1000 iteration and 50 steps (Figure S2B). The number of total ASVs decreased from 25,274 to 24,389 (885 ASVs) and the number of reads per sample decreased between 4,679 and 65,321 (Table S2).

To assess within sample microbial alpha diversity, Chao1 and Shannon's indices were calculated using the *phyloseq* package with the *plot_richness*() function (v1.44.0; McMurdie and Holmes 2013). The significant differences in Chao1 and Shannon were calculated using a *t*‐test and adjusted using Bonferroni correction. Principal Coordinate Analysis (PCoA; Gower 1966) and non‐metric multidimensional scaling (NMDS; Minchin 1987) were used to identify variables explaining the most variation in each sample, enabling the visualisation of sample dissimilarities using the same package. The phylogenetic diversity (PD) of the microbial community was assessed using the *Ses.PD*() function from the *picante* package (v1.8.2; Kembel et al. 2010). To investigate the correlations of environmental variables with alpha and phylogenetic diversity, generalised additive models (GAMs) were employed using the *gam*() function from the *mgcv* package (v1.8–42; Wood 2023).

Principal Component Analysis (PCA; Hotelling 1933) for the geochemistry data was calculated using the *prcom*() function from the *stats* package (R Core Team 2022) and plotted using *ggbiplot* package (v0.55; Vu 2011). The squared correlation coefficient (*r* ^2^) and *p* value were calculated using PERMANOVA with the *adonis2*() function from the *vegan* package (v2.6–4; Oksanen et al. 2015). Additionally, correlation among the geochemical variables was calculated using the *cor*() function (*method = ‘spearman’*) from the *stat*s package, and correlations between the geochemical parameters and microbial communities were assessed using the *envfit*() function from the *vegan* package (v2.6–4; Oksanen et al. 2015). This analysis allowed for the identification of significant correlations between the physicochemical factors and the composition of the microbial communities. To further investigate the differences in microbial communities, we conducted Linear Discriminant Analysis (LDA) to differentiate microbial communities across pond types and identifying biomarkers using *microbiomeMarker* package (v1.10.0; Cao et al. 2022). The function to calculate LDA was *run‐lefse*() with an LDA score of 4, Wilcoxon cutoff of 0.05, and normalisation method of ‘CPM’ (pre‐sample normalisation of the sum of the values to 1e + 06.) These results were plotted using the *plot_abundance*() and *plot_heatmap*() functions from the same package. All other graphs were plotted using the *ggplot2*, *grid*, *corrplot*, and *lattice* packages (Sarkar 2008; Wei and Simko 2021; Wickham et al. 2016). All statistical tests were considered significant at *p* value ≤ 0.05.

## 3 Results and Discussion

### 3.1 Water Chemistry in SDNWA Wetland Ponds

Our data confirmed that our prairie wetland ponds exhibit substantial geochemical heterogeneity, indicating variability across the studied sites (Table 1). Geochemical heterogeneity, in this context, refers to the considerable variations observed in key parameters such as SO_4_^2−^ concentrations (ranging from 37.02 to 14,333.65 mg/L), DOC concentrations (ranging from 9.8 to 168.8 mg/L), and conductivity (ranging from 425 to 13,699 μs/cm). These variations exhibit patterns among the upland and lowland ecosites; ponds with high [SO_4_^2−^] and [DOC] were located in the lowland ecosite and ponds with low [SO_4_^2−^] and [DOC] were in the upland ecosite. Because ponds have limited groundwater inputs (Hayashi, van der Kamp, and Rosenberry 2016), elevated concentrations of DOC and solutes in lowland sites are likely due to watershed inputs over the millenniums of pond evolution. The dugout had the lowest concentrations of both, likely due to the removal of accumulated OM and solutes during construction. These findings are similar to previous studies that classified prairie wetlands into three similar groups based on salinity and topography (Hoggarth, Hall, and Mitchell 2015; Lissey 1968).

**TABLE 1:** Mean geochemistry variables (± standard error of the mean (SE)) in eight ponds and three pond types during the open water season of 2021 (total *n* = 48). SO_4_^2−^ = sulfate concentrations, DOC = dissolved organic carbon concentrations, Temp = temperature, Chl.a = chlorophyll a, ORP = oxidation‐reduction potential.

Pond type	Pond	SO_4_^2−^ mg/L (mean ± SE)	DOC mg/L (mean ± SE)	Conductivity μS/cm (mean ± SE)	pH (mean ± SE)	Temp (°C) (mean ± SE)	Chl.a μg/L (mean ± SE)	ORP (mV) (mean ± SE)
High DOC & SO4	P02	13175.5 ± 1158.0	153.2 ± 15.6	8764.0 ± 4935.0	8.9 ± 0.7	19.5 ± 3.1	71.2 ± 26.2	105.6 ± 0.9
	P35	8668.3 ± 1240.5	179.8 ± 36.9	9923.5 ± 934.9	8.8 ± 0.3	21.7 ± 0.5	131.9 ± 54.2	7.6 ± 40.3
	P97	6089.1 ± 1001.5	114.4 ± 20.1	6742.6 ± 948.6	8.6 ± 0.2	19.9 ± 1.5	56.3 ± 13.1	9.1 ± 33.3
	P124	8413.5 ± 1400.0	117.0 ± 12.1	8788.5 ± 1188.5	8.9 ± 0.3	19.7 ± 1.8	12.3 ± 5.8	26.4 ± 41.7
	P125	4975.5 ± 347.1	83.6 ± 6.0	6143.9 ± 345.3	8.9 ± 0.1	20.2 ± 1.5	240.9 ± 57.5	8.9 ± 31.1
Low DOC & SO4	P109	1719.0 ± 235.2	35.5 ± 3.6	2436.2 ± 129.9	8.7 ± 0.4	22.7 ± 2.3	17.4 ± 9.5	39.2 ± 28.7
	P118	650.1 ± 109.5	38.1 ± 0.8	1583.5 ± 34.5	8.3 ± 0.5	23.9 ± 1.1	37.9 ± 33.3	20.7 ± 4.3
Dugout	P15	44.1 ± 2.8	14.1 ± 1.2	501.8 ± 106.4	9.4 ± 0.2	18.9 ± 1.2	89.5 ± 53.2	28.3 ± 20.1

**FIGURE 2:**
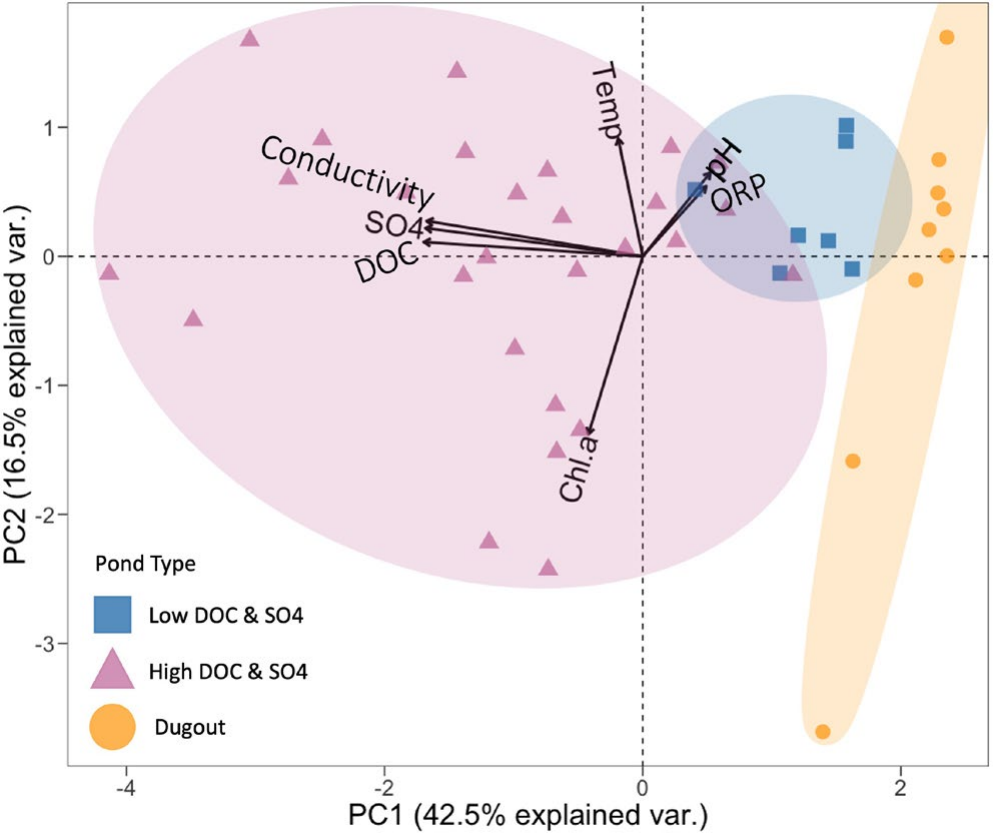
Principal Component Analysis (PCA) biplot of geochemical variables across three pond types during the open water season 2021. Each arrow represents a geochemical variable; its direction indicates the variable's contribution to the principal components. The points represent different samples from a pond (*n* = 48), colour/shape coded by the pond type (Low DOC & SO4 in blue/square, High DOC & SO4 in pink/triangle, Dugout in orange/circle). Ellipses were calculated based on Khachiyan's algorithm using the *ggforce* package (v0.4.2; Pedersen 2024). SO_4_^2−^ = Sulfate concentration (mg/L), DOC = Dissolved organic carbon concentration (mg/L), Conductivity (μS/cm), Temp = Temperature (°C), Chl.a = Chlorophyll a (μg/L), ORP = Oxidation reduction potential (mV).

Sulfate and DOC concentrations had a strong positive correlation to one another, a negative correlation to pH and oxidation‐reduction potential (ORP), and a weak positive correlation to Temp and Chl.a (Figure S3). The correlation between pH and ORP was not significant, and the pH and Temp showed a weak negative correlation (Figure S3). Three pond types were differentiated by PCA analysis based on their geochemical parameters, with the first two components (PC1 and PC2) explaining more than half of the variation in the geochemical data (Figure 2; Table S3). The first component (PC1) was mainly weighted by [SO_4_^2−^] and [DOC] and conductivity, while pH, Temp, Chl.a, and ORP influenced PC2 (Table S3). Ponds are herein referred to as ‘High DOC & SO4’ (ponds with concentrations of DOC > 65.85 mg/L and SO_4_^2−^ > 3347.25 mg/L; located in the lowland ecosite) and ‘Low DOC & SO4’ (ponds with concentrations of DOC < 65.85 mg/L and SO_4_^2−^ < 3347.25 mg/L, located in the upland ecosite) based on the 25th percentile of the measured concentrations (Table S4). This classification was further supported by statistical analysis using a *t*‐test, which confirmed that differences in concentrations between the ‘High’ and ‘Low’ groups were statistically significant (*p* value = 0.0059 for [SO_4_^2−^] and 0.0050 for [DOC]; Table S4). The dugout is not included in these groups because we had only one dugout and therefore kept it separate and herein, we are referring to it as the ‘Dugout.’ These pond types were consistent with previous studies (Hall et al. 2023; Hall, Baron, and Somers 2009; Hoggarth, Hall, and Mitchell 2015; Tran, Vu, and Hall 2022).

Our work confirmed a number of relationships found in previous studies in the SDNWA (Waiser 2006; Bates and Hall 2012; Hoggarth, Hall, and Mitchell 2015; Hall et al. 2023), primarily the strong positive correlation between [SO_4_^2−^] and [DOC] which facilitated the categorization of our ponds based on high and low concentrations of both parameters (Figure S3). This relationship between [DOC] and [SO_4_^2−^] in other wetland systems has been inconsistent; positive (e.g., paddy soil Chen et al. 2020), negative (e.g., wetland‐draining stream Eimers et al. 2008), and neutral correlations (e.g., peat‐covered catchment Worrall, Burt, and Adamson 2008) have been found in various wetland complexes. This speaks to the heterogenic nature of wetlands systems. The relationships of pH and ORP with temperature showed weak positive correlations, while they exhibited negative correlations with all other parameters. Chl.a showed positive correlations with [SO_4_^2−^], [DOC], and conductivity.

As expected, we also confirmed the strong positive correlation between conductivity and SO_4_^2−^ concentrations due to dissolved SO_4_^2−^ ions being the main ion that contributes to the overall conductivity in many of these ponds. Differences observed across various wetland and freshwater environment systems may be due to the fact that the microbial SO_4_^2−^ reduction is primarily controlled by SO_4_^2−^ concentration, facilitating SO_4_^2−^ reduction over CO_2_ reduction and limiting carbon mineralization until SO_4_^2−^ is depleted (Chen et al. 2020). In addition, sulfate‐reducing bacteria are able to use SO_4_^2−^ as their electron acceptor to degrade organic compounds, contributing to increased DOC in wetlands (Muyzer and Stams 2008). Our lowest DOC concentration (15 mg/L) is comparable to the mean values found in various systems, despite our categorization of wetland ponds based on low to high DOC concentrations. In fact, the mean DOC concentration of our ponds (90 mg/L) was much higher than the mean DOC concentration in seawater (0.5 mg/L), groundwater (0.7 mg/L), oligotrophic lakes (2 mg/L), rivers (5 mg/L), eutrophic lakes (10 mg/L), marsh ecosystems (15 mg/L), and bogs (30 mg/L) as reported by Thurman (1985).

### 3.2 Microbial Community Composition in SDNWA Wetland Ponds

**FIGURE 3:**
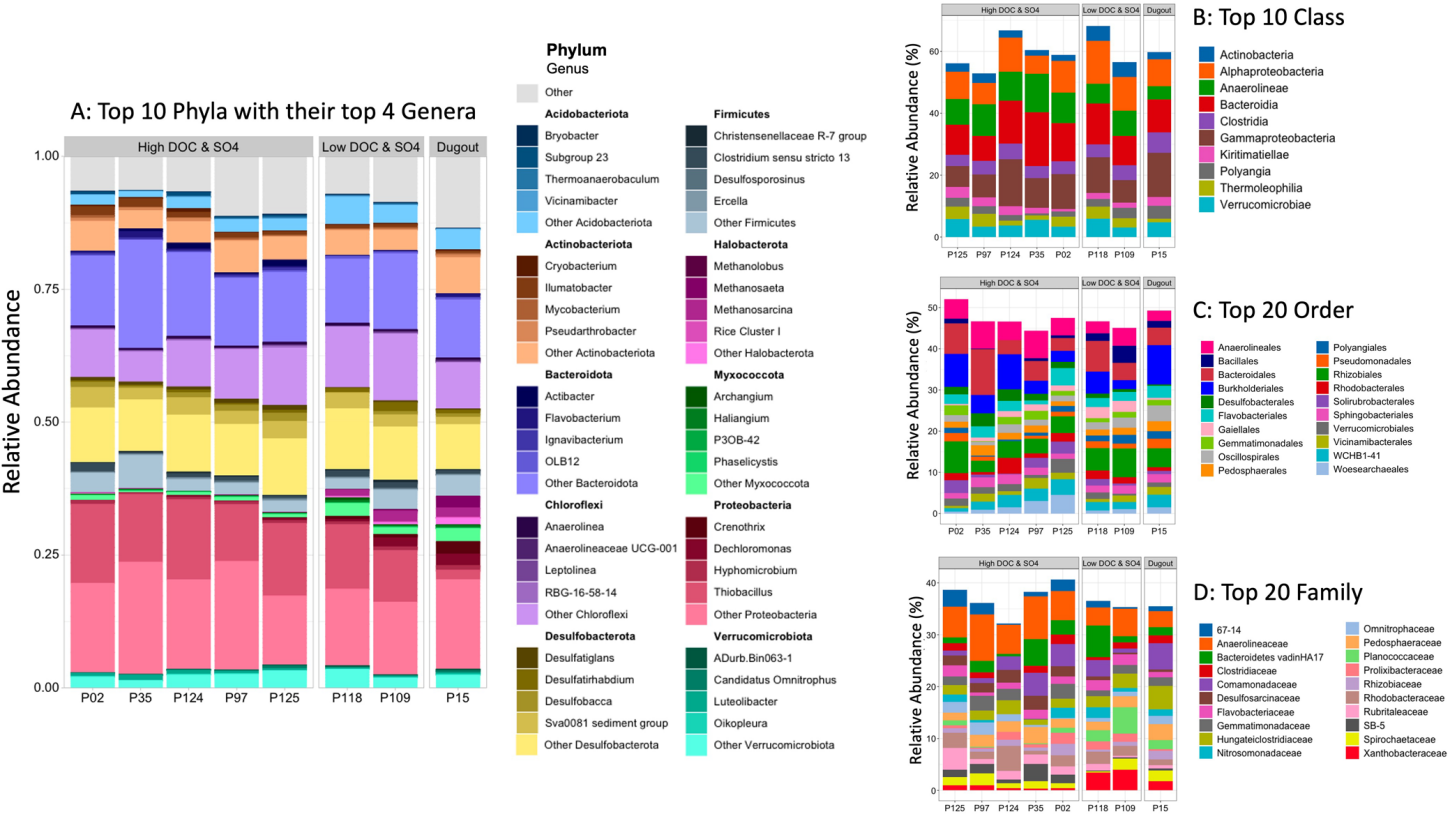
Relative abundance of microbial communities across different taxonomic levels based on 16S rRNA gene analysis and identified using the SILVA V138 database. (A) top 10 phyla and their top 4 genera, (B) top 10 classes, (C) top 20 orders, and (D) top 20 families. Each bar represents the cumulative microbial community composition of all samples from an individual pond, with taxa colour‐coded according to their classification. The legends in each panel provide taxonomic references specific to the respective taxonomic level presented.

The microbial communities showed differences among pond types. The microbial communities were composed of 72 phyla across all samples, with several dominant phyla consistently observed in all the wetlands. The two most abundant phyla as a proportion of total phyla in all ponds were *Proteobacteria* (High DOC & SO4: ~32%, Low DOC & SO4: ~27%, and Dugout ~25%) and *Desulfobacterota* (High DOC & SO4: ~17%, Low DOC & SO4: ~15%, and Dugout ~14%; Figure 3A). In High DOC & SO4 ponds, the next most abundant phyla included *Bacteroidota* (~20%), *Chloroflexi* (~10%), *Firmicutes* (~7.5%), and *Actinobacteriota* (~7%) while, in Low DOC & SO4 ponds, the next most abundant phyla were *Chloroflexi* (~12%), *Bacteroidota* (~11%), and *Acidobacteriota* (~7%). In the Dugout, the next most abundant phyla were *Bacteroidota* (~12%) and *Actinobacteriota* (~6%), and two phyla (that were of very low abundance in High DOC & SO4 and Low DOC & SO4 ponds) from the Archaea Kingdom: *Halobacterota* (~5%) and *Euryarchaeota* (~3%).

#### 3.2.1 Heterogeneity of Microbial Community Composition in SDNWA Wetland Ponds

**FIGURE 4:**
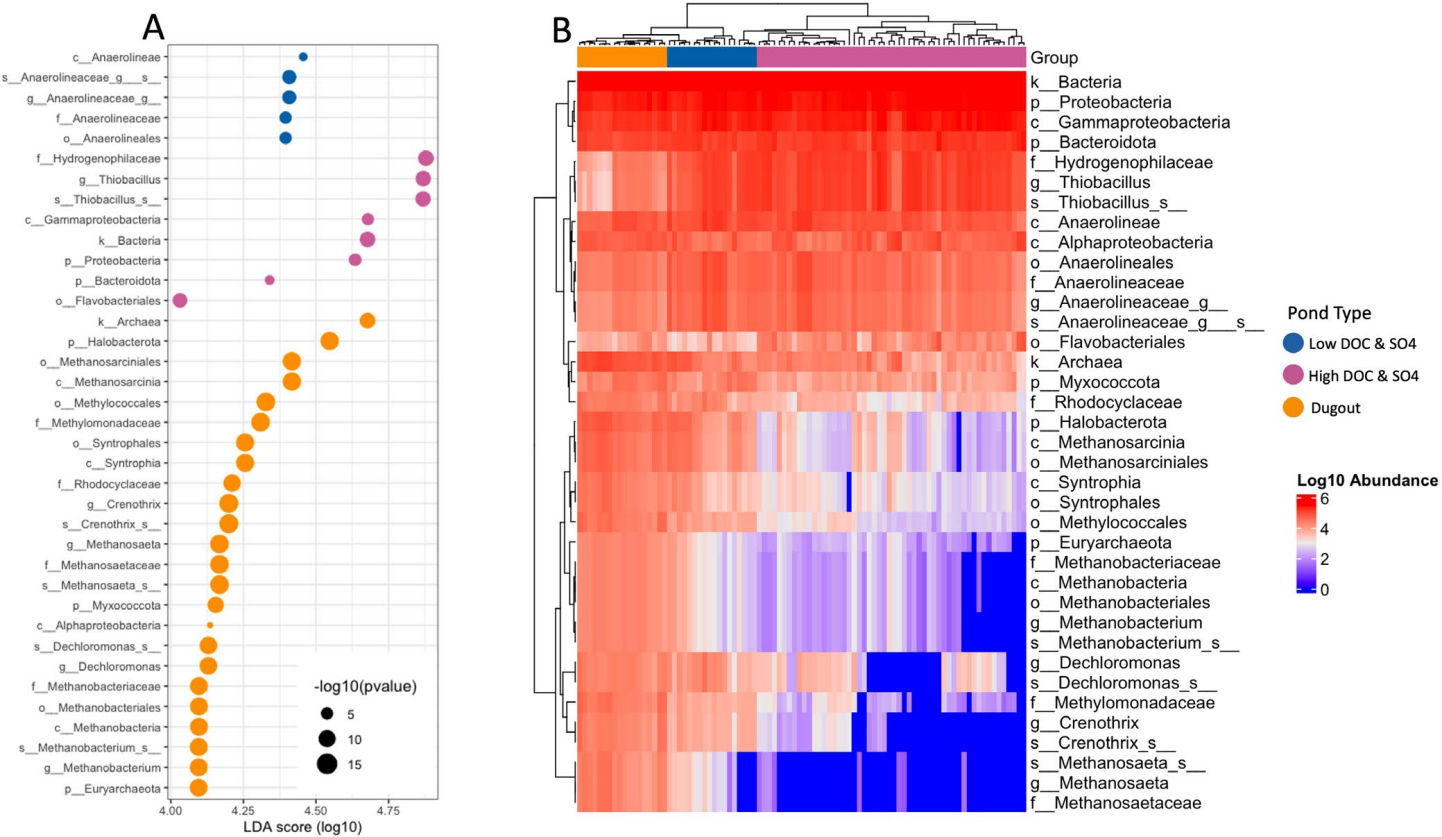
Differentially abundant taxa across different pond types identified using Linear Discriminant Analysis (LDA) Effect Size (LEfSe). (A) The dot plot displays the effect sizes (LDA scores) of taxa that are significantly different (LDA score > 4, *p* < 0.05) among the pond types: Dugout (orange), Low DOC & SO4 (blue), and High DOC & SO4 (pink). Larger dots represent taxa with higher relative abundance differences between groups. (B) Heatmap of differentially abundant taxa across pond types, clustered by taxa and pond types. The heatmap illustrates the relative abundance of significant taxa identified by LEfSe analysis (LDA score > 4, *p* < 0.05) in Dugout (orange), Low DOC & SO4 (blue), and High DOC & SO4 (pink). Hierarchical clustering was applied to taxa, representing patterns of microbial community composition across different pond types.

In our wetlands, sulfur‐cycling (*Proteobacteria, Desulfobacterota*) and carbon‐cycling (*Bacteroidota, Firmicutes*) microorganisms were the most prominent taxa (Figure 3A). The geochemical conditions of the ponds likely impacted the microbial distribution and potentially modulated their functional roles within the ecosystem. In ponds characterised by high [SO_4_^2−^] and [DOC], *Proteobacteria*, particularly its sulfate‐metabolising members (such as genus *Thiobacillus* and order *Rhizobiales*; Konrad et al. 2023; Figure 3A,D), were more abundant alongside *Desulfobacterota's* sulfur‐reducing members (such as order *Desulfobacterales, and genus Desulfatiglans*), crucial for SO_4_^2−^ reduction processes (Ci et al. 2022; Jochum et al. 2018; Kushkevych et al. 2021; Zhao et al. 2023, Figure 3C,D). In these ponds, *Bacteroidota* were also abundant and they, along with *Firmicutes* contribute to the organic matter decomposition (Huang et al. 2023; Zhang et al. 2022). The abundance of methanogens (methanogenic members of *Halobacterota* and *Euryarchaeota*; Table S5) was low in High DOC & SO4 ponds, likely due to competition from SO_4_^2−^ reducers (Kristjansson, Schönheit, and Thauer 1982). To investigate this further, we conducted Linear Discriminant Analysis (LDA) to differentiate microbial communities across pond conditions. The LDA results revealed that methanogens, specifically from the methanogenic members of *Halobacterota* (order *Methanosarciniales*; Kendall and Boone 2006) and *Euryarchaeota* (order *Methanobacteriales*; Bonin and Boone 2006), were significantly more abundant in Low DOC & SO4 and Dugout ponds compared to High DOC & SO4 ponds (Figure 4A). Additionally, a heatmap to visualise the relative abundance of differentially abundant taxa across the pond types confirmed that methanogens were more prevalent in Low DOC & SO4 and Dugout ponds, clustering distinctly from the microbial communities in High DOC & SO4 ponds (Figure 4B). This might be due to limited SO_4_^2−^ availability (therefore giving an advantage to methanogens over sulfate‐reducing bacteria) and their ability to use inorganic carbon and simple organic compounds for methane production (La et al. 2022).

#### 3.2.2 The Potential Impact of Microbial Communities on Other Biogeochemical Cycles in the SDNWA Wetland Ponds

The microbial communities in the SDNWA play roles beyond carbon and sulfur cycling, influencing other biogeochemical cycles as well. For instance, nearly all sulfur‐reducing bacteria, such as those within the phylum *Desulfobacterota*, are capable of transforming mercury (Hg) into methylmercury (MeHg), a neurotoxin that can bioaccumulate in food chains, posing risks to wildlife and humans (Capo et al. 2022; Peterson et al. 2020). One of the abundant taxa identified as a biomarker for High DOC & SO4 ponds in the LDA results (Figure 4A) was *Gammaproteobacteria*, a class associated with the phosphorus (P) cycle. A study in the coastal wetlands of China found that *Gammaproteobacteria* abundance was higher in brackish wetlands than in freshwater ones, suggesting its role in P bioavailability under varying salinity conditions (Hu et al. 2023). Additionally, phyla like *Chloroflexi* and its family *Anaerolineaceae* can decrease P bioavailability by immobilising it (Wang et al. 2021). This family was more abundant in Low DOC & SO4 and was also highlighted in the LDA results for this pond type (Figure 4A). Interestingly, after methanogens, another biomarker for the Dugout was the genus *Crenothrix*, a known methane consumer that may impact methane cycling processes (in stratified lakes: Oswald et al. 2017; in shallow lakes: Yang et al. 2022).

### 3.3 Microbial Community Alpha Diversity and Phylogenetic Diversity in Three Pond Types

**FIGURE 5:**
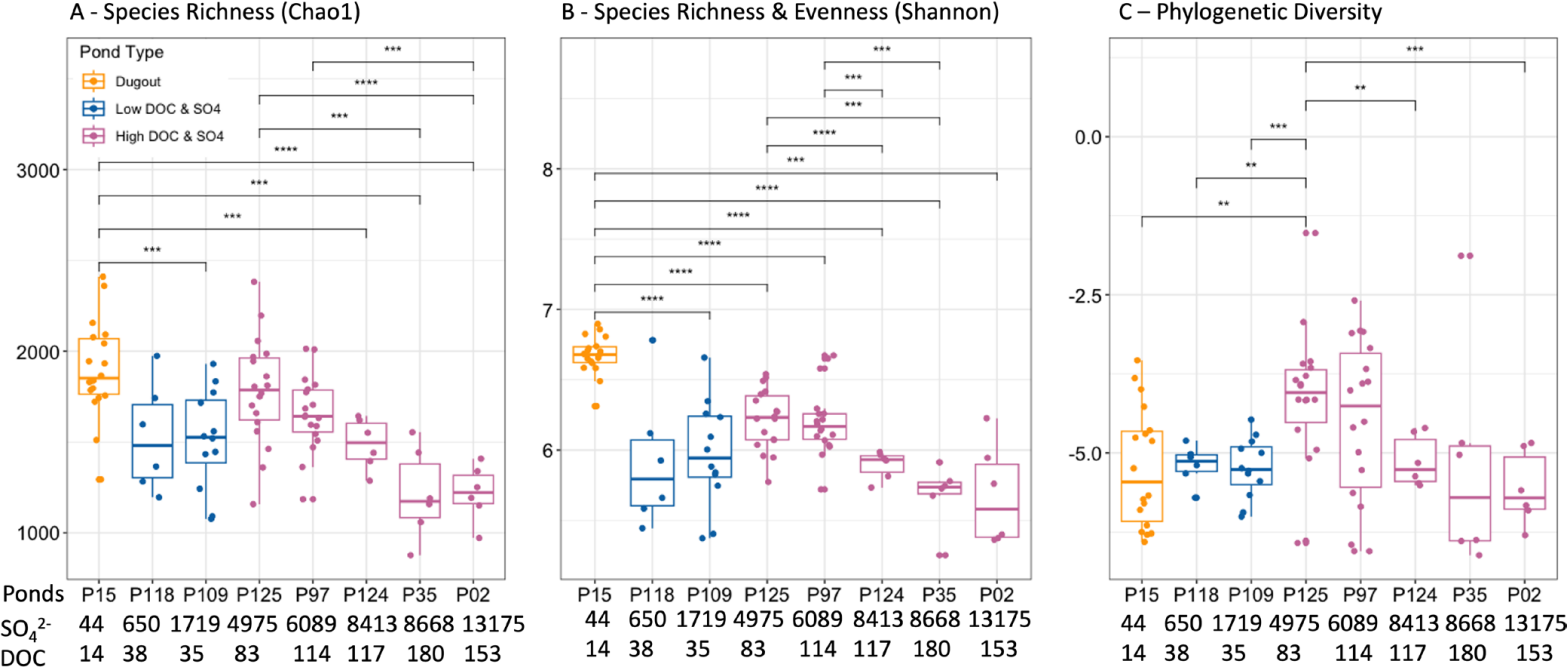
Alpha and phylogenetic diversity analysis based on the total number of species derived from 16S rRNA gene‐based sequencing of each pond type during the open water season 2021. (A, B) Species richness and evenness (using Chao1 and Shannon's indices) were calculated using the *phyloseq* package to find the alpha diversity. (C) Phylogenetic diversity (using Ses.PD) was calculated using the *picante* package to find phylogenetic diversity (*n* = 90). Each box plot shows the smallest (lower box) and largest (upper box) values for the first and third quartiles (25% and 75%), the medians (middle line), the upper and lower whisker (no further than 1.5 inter‐quartile range) and points beyond whiskers (outliers). Each colour represents a pond type (Low DOC & SO4 in blue, High DOC & SO4 in pink, Dugout in orange). SO_4_^2−^ = Mean sulfate concentrations (mg/L), DOC = Mean dissolved organic carbon concentrations (mg/L), Ses.PD = Standardised effect size of phylogenetic diversity. Significance levels: ****p < 0.0001, ***p < 0.001, **p < 0.01, *p < 0.05, calculated by *t*‐test and adjusted using Bonferroni correction. *Note:* Non‐significant comparisons are not shown in the figure.

Species richness (using the Chao1 index/alpha diversity; Hughes et al. 2001) in the Dugout was significantly higher than in all other ponds (*p* value ≤ 0.05) with the exception of P118, P125, and P97 where it was similar (*p* value > 0.05; Figure 5A; Table S6; Figure S4). The species richness of all High DOC & SO4 ponds were similar to one another with the exception of P35 and P02 which were significantly lower than in P125 and P97 (*p* value > 0.05; Figure 5A; Table S6; Figure S4). Species richness and evenness (using Shannon index/alpha diversity; Feranchuk et al. 2018) were significantly higher in the Dugout compared to all other ponds (*p* value ≤ 0.05; Figure 5B). In the High DOC & SO4 ponds, P125 and P97 had significantly higher alpha diversity compared to P124 and P35 (*p* value ≤ 0.05). There were no differences in richness and evenness in the Low DOC & SO4 pond group (*p* value > 0.05). The standardised effect size of phylogenetic diversity (using Ses.PD; Kembel et al. 2010) was significantly higher in P125 compared to most ponds (*p* value ≤ 0.01), except in P97 and P35 where it was similar (*p* value > 0.05; Figure 5C; Table S6, Figure S4). The correlations of richness and phylogenetic diversity based on GAMs with [DOC] and [SO_4_^2−^] were non‐linear but strong (*r* ^2^ > 0.5, *p* value < 0.05; Figure S5). The correlation of richness with pH and Temp was linear but relatively weak (*r* ^2^ = 0.0365, *r* ^2^ = 0.00302, *p* value < 0.05 respectively; Figure S6) compared to [DOC] and [SO_4_^2−^]. The correlations of phylogenetic diversity with pH and Temp were not linear, very weak with pH (*r* ^2^ = 0.0079, *p* value < 0.05), and relatively weak with Temp (*r* ^2^ = 0.213, *p* value < 0.05; Figure S6). For rarefied data, similar trends were observed (Figure S4A–C). Richness and evenness assess the number of species and distribution of abundance of species in an environment (Willis 2019) while high phylogenetic diversity implies a broader range of genetic and ecological differences among species, indicating a suitable habitat for various types of microorganisms, and low phylogenetic diversity suggests a more limited set of closely related species, pointing toward a suitable habitat for fewer taxa (Faith 1992).

#### 3.3.1 Can the Heterogeneity of Microbial Community Composition Be Explained by the Heterogeneity of DOC and SO_4_^2^ ^−^ Concentrations in Water?

Although not linear, we did find strong correlations between various DOC and SO_4_^2−^ concentrations and microbial communities' alpha diversity (both richness and evenness of taxa) and phylogenetic diversity (evolutionary relationship among taxa; Figure 5, Figure S5). In sediments within ponds characterised by low concentrations of DOC (10–18 mg/L) and SO_4_^2−^ (37–58 mg/L), we observed high microbial alpha diversity, alongside reduced phylogenetic diversity. With increasing DOC and SO_4_^2−^ concentrations (inflection point at 30–48 mg/L and 540–2,600 mg/L for [DOC] and [SO_4_^2−^], respectively alpha diversity decreased without a corresponding decline in phylogenetic diversity). The peak values for both alpha diversity and phylogenetic diversity were observed within the ranges of 56–115 mg/L and 5,000–6,000 mg/L for [DOC] and [SO_4_^2−^], respectively. Subsequently, we observed a decrease in both parameters in ponds characterised by concentrations of 150–180 mg/L for [DOC] and 8,000–14,000 mg/L for [SO_4_^2−^]. We suggest the existence of a ‘sweet spot’ for richness/evenness and phylogenetic diversity at moderate [DOC] and [SO_4_^2−^] which is an indication that moderate concentrations of DOC and SO_4_^2−^ suit a diverse microbial community without creating selection pressures favouring specific taxa.

### 3.4 Dissimilarity of Microbial Community Composition in Three Pond Types

**FIGURE 6:**
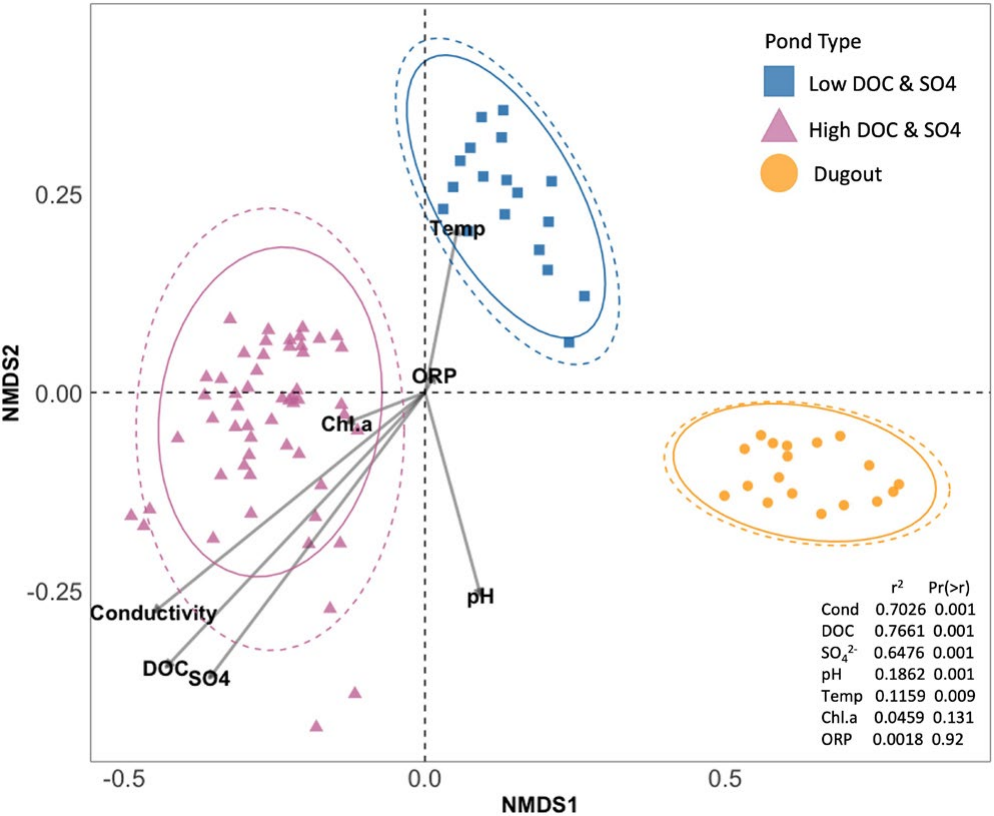
Non‐metric multidimensional scaling (NMDS) of microbial community composition and geochemical variables (*n* = 90). Points represent individual samples, and each colour/shape represents a pond type (Low DOC & SO4 = blue/square, High DOC & SO4 = pink/triangle, Dugout = orange/circle). Ellipses represent 95% confidence intervals around the centroid (solid line = t distribution; dashed line = normal distribution). The arrows represent the geochemistry data based on the *envfit()* function (with permutations = 999) that fits geochemistry data onto the ordination plot from the *vegan* package (v2.6–4; Oksanen et al. 2015). Short arrows indicate weak predictors and long arrows indicate strong predictors. The small table on the right shows the goodness of fit statistic (squared correlation coefficient; *r* ^2^) and the probability (*p* value) of *r* ^2^ (using the *vegan* package, v2.6–4; Oksanen et al. 2015). SO_4_^2−^ = Sulfate concentration (mg/L), DOC = Dissolved organic carbon concentration (mg/L), Conductivity (μS/cm), Temp = Temperature (°C), Chl.a = Chlorophyll a (μg/L), ORP = Oxidation reduction potential (mV).

Ponds with different DOC and SO_4_^2−^ concentrations were dissimilar in their composition as evidenced by the Bray‐Curtis distance and PCoA method among the three pond types (Figure S7). Axis 1 and 2 accounted for approximately 26% and 9% of the microbial community composition variation respectively, based on pond type, demonstrating a clear separation of microbial communities when categorised by pond type (PERMANOVA: *r* ^2^ = 0.33, *p* value < 0.001; Figure S7). Furthermore, when correlating the microbial communities in all pond types with the environmental variables, we observed that microbial community composition was highly correlated with DOC and SO_4_^2−^ concentrations and conductivity (*r* ^2^ = 0.76, 0.70, and 0.64 respectively; *p* value < 0.05; Figure 6), but not correlated with ORP and Chl.a (*r* ^2^ = 0.0018 and 0.0459 respectively; *p* value > 0.05; Figure 6). When considering ordination within each pond type, in the High DOC & SO_4_^2−^ ponds, microbial communities were positively correlated with conductivity, DOC and SO_4_^2−^ concentrations and negatively correlated to Temp and pH, while communities in Low DOC & SO4 ponds were negatively correlated with [DOC] and [SO_4_^2−^] and positively correlated with Temp (Figure 6). The microbial community composition in the Dugout was negatively correlated to all the geochemical variables except pH where the correlation was positive.

#### 3.4.1 Why Are Microbial Community Composition Correlations With DOC and SO_4_^2^ ^−^ Concentrations Not Linear?

We expected that high [DOC] would promote high species richness and phylogenetic diversity, moderate [DOC] would promote moderate richness and phylogenetic diversity, and low [DOC] would correspond to low richness and phylogenetic diversity because diversity is, in a large part, controlled by the availability of nutrients and energy sources. The trends we observed do not fit the expectation that higher DOC concentrations in water relate to higher phylogenetic diversity and richness (Bending, Turner, and Jones 2002; Li et al. 2018; Staddon, Duchesne, and Trevors 1997). We observed the opposite pattern: lower richness occurred in ponds with the highest [DOC], while ponds with the lowest [DOC] and ponds with moderate [DOC] exhibited higher richness (Figure S4, Table 2). We also observed a bell‐curved trend for phylogenetic diversity versus DOC concentrations (Figure S4); the highest phylogenetic diversity was observed in ponds with moderate [DOC], whereas in all other ponds (with high and low [DOC]) phylogenetic diversity was low (Figure S4, Table 2). The unexpected trend of high [DOC] related to low alpha and phylogenetic diversity suggests a potential inhibitory effect on diversity/richness/evenness at high [DOC] because high [DOC] might be favourable to specific taxa able to use DOC at high concentrations while out‐competing other taxa.

**TABLE 2:** Corresponding patterns among sulfate concentration (SO_4_^2−^), dissolved organic carbon concentration (DOC), alpha diversity (richness and evenness), and phylogenetic diversity of microbial communities in ponds.

SO_4_^2−^ (mg/L)	DOC (mg/L)	Richness and evenness	Phylogenetic diversity
Very low (37–58)	Very low (10–18)	High	Low
Low (540–2,600)	Low (30–48)	Low	Low
Moderate (5,000–6,000)	Moderate (56–115)	High	High
High (8,000–14,000)	High (150–180)	Low	Low

In our ponds, DOC concentrations are correlated with the optical quality of DOC (Khan, et al., submitted), suggesting DOC origin may be key to predicting the microbial community shifts in an environment. For example, in Arctic coastal rivers, species of the phyla *Proteobacteria*, *Bacteroidetes*, *Actinobacteria*, and *Verrucomicrobia* were found to flourish in high‐nutrient environments with high concentrations of terrestrially derived dissolved organic matter (Sipler et al. 2017). Similarly, a laboratory assay on more than 200 soil samples from various ecosystem types (grassland, woodland, agricultural, and pine forest) observed low microbial richness at high DOC concentrations and vice versa (Albright et al. 2020). Albright et al. (2020) also examined DOC quality and suggested that microbial communities alter the quality of DOC in high DOC conditions (enriching DOC with more mineral binding potential) which in turn might impact the abundance because some taxa can use enriched DOC, while other microbes might have lower carbon storage potential and therefore cannot thrive in enriched DOC.

The suitable quality and quantity of DOC alone may not explain why an environment is favourable for certain species to thrive. We observed a positive correlation between [DOC] and [SO_4_^2−^] and strong correlations between these two variables and the microbial community composition (Figure S4). Therefore, it is important to consider the possible influence of SO_4_^2−^ on microbial communities, as it may play a significant role, alongside DOC, in shaping community composition. Sediments from a seasonally flooded urban freshwater wetland Wang et al. (2024) amended with SO_4_^2−^ had significantly altered microbial communities and metabolic processes, such as methane production and carbon cycling. This shift can be explained by the redox ladder, where sulfate reduction is thermodynamically favoured over methanogenesis, allowing sulfate‐reducing bacteria to outcompete methanogens in the presence of high [SO_4_^2−^] (Conrad 1999). Methanogens rely on a limited set of substrates, primarily acetate (acetoclastic methanogens) and H_2_/CO_2_ (hydrogenotrophic methanogens), with H_2_ concentrations typically remaining low due to rapid turnover in anaerobic environments (Enrich‐Prast, Bastviken, and Crill 2009). This low H_2_ availability is important for syntrophic interactions between H_2_‐producing bacteria and H_2_‐consuming methanogens (Conrad 1999). However, when SO_4_^2−^ is present, sulfate reducers can efficiently consume H_2_, leaving less available for methanogens and thereby suppressing methanogenic activity (Conrad 1999). This results in reduced methane emissions and shifts carbon metabolism toward sulfate reduction, favouring sulfate‐reducing bacteria over methanogens. This dynamic can lead to shifts in microbial guilds and reduce overall diversity (Wang et al. 2024). Therefore, while high [DOC] suppresses the overall richness due to the competitive advantage gained by specialised microbes, in sediments of ponds with the highest [SO_4_^2−^], these microbes may thrive at the expense of other species creating a niche while suppressing overall phylogenetic diversity (Table 2). The Dugout with the lowest DOC and SO_4_^2−^ concentrations had the highest richness and low phylogenetic diversity. Here, the two secondary dominant phyla, *Halobacterota* (orders *Methanosarciniales and Methanomicrobiales*) and *Euryarchaeota* (order *Methanobacteriales*) are methanogens that use inorganic carbon as a source of energy (Table S5; Zhang et al. 2023) and their diversity might be negatively correlated with DOC as it was shown in various wetlands in China (Liu et al. 2012). In addition, this pond had the lowest SO_4_^2−^ concentration, which influences microbial competition by limiting the availability of electron acceptors that occupy a higher position on the redox tower. In anaerobic environments, microbes use available electron acceptors in a sequence based on their redox potential, with higher‐potential acceptors, such as SO_4_^2−^, used before lower‐potential acceptors, such as CO_2_ (Conrad 1999). Sulfate‐reducing bacteria typically outcompete methanogens when SO_4_^2−^ is abundant; however, when [SO_4_^2−^] are low, hydrogenotrophic methanogens gain a competitive advantage because they can use CO_2_ as an electron acceptor, which lies lower on the redox tower (Dar et al. 2008). This lack of competition from sulfate reducers allows methanogenic activity to dominate, leading to increased methane production in sulfate‐poor environments (Koebsch et al. 2019). Therefore, while DOC might be a limiting nutrient for other taxa in this pond (hence the low phylogenetic diversity), some specific taxa like methanogens that use inorganic carbon (mainly hydrogenotrophic methanogens; Table S5) may thrive (hence the high richness; Table 2). This observed ‘sweet spot’ of having both high richness and high phylogenetic diversity was observed in the moderate concentration of DOC in the same ponds with a moderate concentration of SO_4_^2−^ (Table 2) suggesting that in those ponds the concentration of DOC was not high enough to create a niche for some specific taxa and the concentration of SO_4_^2−^ was not high enough to create a selection pressure (hence the high richness and phylogenetic diversity).

Although we identified statistically significant differences in phylogenetic diversity among ponds (*p* value < 0.05), all phylogenetic diversity values were negative (with low quantiles), signifying small phylogenetic distances (Kembel et al. 2010) and suggesting a selection pressure leading to lower phylogenetic turnover (Martiny et al. 2015). Lower phylogenetic turnover implies that environmental conditions or other factors favour the persistence of closely related species rather than promoting diversity. However, whether this selection originates from high or low DOC and SO_4_^2−^ concentrations remains unclear, necessitating further investigation to validate this hypothesis.

### 3.5 How Do Other Environmental Variables Influence Microbial Community Composition?

While other studies have highlighted the influence of pH (e.g., wetland soil by Hartman et al. 2008; alpine wetland soil by Kang et al. 2021) and temperature (e.g., arctic lakes and streams by Adams, Crump, and Kling 2010; sub‐Antarctic freshwater by Lavergne et al. 2021; freshwater lakes by Shade, Jones, and McMahon 2008) on microbial communities, our ordination analysis and GAMs showed weaker correlations between these two parameters and microbial communities compared to DOC and SO_4_^2−^ concentrations (Figure S5, Table 2). Weak correlations between pH and temperature and microbial community composition in our study may be attributed to several factors. First, the wide tolerance range of microbial communities to pH and temperature fluctuations within our study area may limit the statistical power to detect patterns and relationships. When variability in one variable, such as pH or temperature, is limited due to microbial tolerance, it can become challenging to find its influence on microbial community composition. Another explanation might be that we did not measure the pH and temperature of the soil, but rather the surface water and therefore, these two parameters might have different values in soil and sediment (due to their buffering capacities) from the surface water. However, some studies have shown that there is a correlation between soil pH and surface water pH in shallow wetlands (Di Luca et al. 2017) and soil temperature and water temperature in shallow lakes (Shinohara, Tsuchiya, and Kohzu 2021). Our ponds, characterised as shallow wetland ponds, often have limited water depth, allowing for interaction between the water column and the underlying sediment (Meerhoff and Jeppesen 2009). As a result, there is increased potential for thermal and chemical exchange between the water and sediment, and therefore water geochemistry might be an easy and low‐cost proxy to determine soil/sediment geochemistry.

Our work showed that variations in microbial community composition within our ponds can be attributed to two key environmental variables, DOC and SO_4_^2−^ concentrations. In the microbial communities of all three pond types, there were a few generalists with a very high abundance and many specialists with various abundance. On one hand, most of these abundant microorganisms play a role in sulfur and carbon cycling (impacting the decomposition of organic matter and SO_4_^2−^ production) while the specialists might impact the quality and availability of DOC and SO_4_^2−^. On the other hand, the various concentrations of DOC and SO_4_^2−^ likely impact these microorganisms, influencing their distribution and potentially modulating their functional roles within the ecosystem.

## 4 Conclusion

Deciphering the correlation between environmental variables and microbial communities' composition of very heterogenic systems like prairie wetlands can help us understand how various changes in the environment might impact the biogeochemical regulators. We found that: (1) the geochemistry of various ponds was different from one another; (2) the microbial communities in different ponds had different compositions; and (3) DOC and SO_4_^2−^ concentrations are key factors that shape microbial community composition.

Microbial communities identified in this study are likely to influence various biogeochemical processes, including methane emission, carbon sequestration, heavy metal transformation, and sulfur species conversion. The dynamics of these microbial communities and their interactions with the environment can significantly impact numerous biogeochemical cycles within these expansive wetland complexes.

In the context of today's rapidly changing environment, where anthropogenic activities and the effects of climate change exert profound influences, understanding microbial community dynamics becomes increasingly crucial. This study, conducted in the wetland ponds of the PPR, provides valuable insights into the relationship between environmental factors and microbial communities and highlights the importance of recognising how microbial communities are adapted to their specific environmental niches thus impacting biogeochemical cycles. Our work on the nuanced dynamics of microbial communities in response to geochemical changes contributes to the broader dialogue on how environmental alterations may influence the functions and stability of ecosystems in the face of ongoing global changes. Recognising thresholds and projecting the consequences of environmental change is important for informed decision‐making in conservation and ecosystem management.

## Author Contributions

**Zohra Zahir:** conceptualization, investigation, writing – original draft, methodology, validation, visualization, writing – review and editing, software, formal analysis, data curation. **Faraz Khan:** methodology, investigation, validation. **Britt D. Hall:** conceptualization, investigation, funding acquisition, methodology, validation, writing – review and editing, project administration, supervision, resources.

## Conflicts of Interest

The authors declare no conflicts of interest.

## Supporting information


Data S1.

## Data Availability

The data that supports the findings of this study are available in the Supporting Information of the manuscript.
