# Supplementary material for: Sulfate and Dissolved Organic Carbon Concentrations Drive Distinct Microbial Community Patterns in Prairie Wetland Ponds
Source: Environ Microbiol Rep. 2025 Jan 27;17(1):e70069. doi: 10.1111/1758-2229.70069 (PMC11772329; doi:10.1111/1758-2229.70069)
Supplement: Supplementary file 1 — Data S1. [file EMI4-17-e70069-s001.pdf]

Supplement Information: Sulfate and Dissolved Organic Carbon Concentrations Drive Distinct Microbial Community Patterns in Prairie Wetland Ponds

Zohra Zahir, Faraz Khan, Britt D. Hall

Department of Biology, University of Regina, 3737 Wascana Parkway, Regina, SK, S4S 0A2 Canada.

Number of tables: 6

Number of figures: 7

Table S1. The GPS coordinates for ponds in St. Denis National Wildlife Area were sampled from May to September 2021. Lowland and Upland are two ecosites based on gradients of slopes and the mechanism of groundwater and runoff recharge and discharge in this area. Dugout is a historical agriculture reservoir which is also located in the Upland ecosite. The pond's permanency is based on the duration of inundation. Permanent wetland ponds have water throughout the year, semi-permanent are inundated for 2-3 months, and ephemeral are inundation for 1-2 months. The area of ponds is based on a rough estimate using Google Earth measuring tools. The depth of the pond is based on the water depth at the time of water and sediment sampling.

				Coordinates of Ponds	
**Pond name**	**Pond Permanency**	**Area (ha)**	**Depth (m)**	**Latitude**	***Longitude***
Lowland					
P97	Semi-permanent	0.41	0.21	*N 52°12'34.8"*	*W 106°04'40.1"*
P125	Semi-permanent	1.07	0.29	*N 52°12'46.5"*	*W 106°04'51.0"*
P02	Ephemeral	0.11	0.10	*N 52°12'53.8"*	*W 106°05'16.2"*
P124	Ephemeral	0.07	0.24	*N 52°12'41.5"*	*W 106°04'48.8"*
P35	Semi-permanent	0.16	0.10	*N 52°12'41.1"*	*W 106°06'19.3"*
Upland					
P15 (Dugout)	Permeant	0.06	0.67	*N 52°12'54.5"*	*W 106°05'41.7"*
P118	Ephemeral	0.02	0.05	*N 52°12'35.3"*	*W 106°05'03.3"*
P109	Semi-permanent	0.16	0.20	*N 52°12'31.5"*	*W 106°05'08.2"*

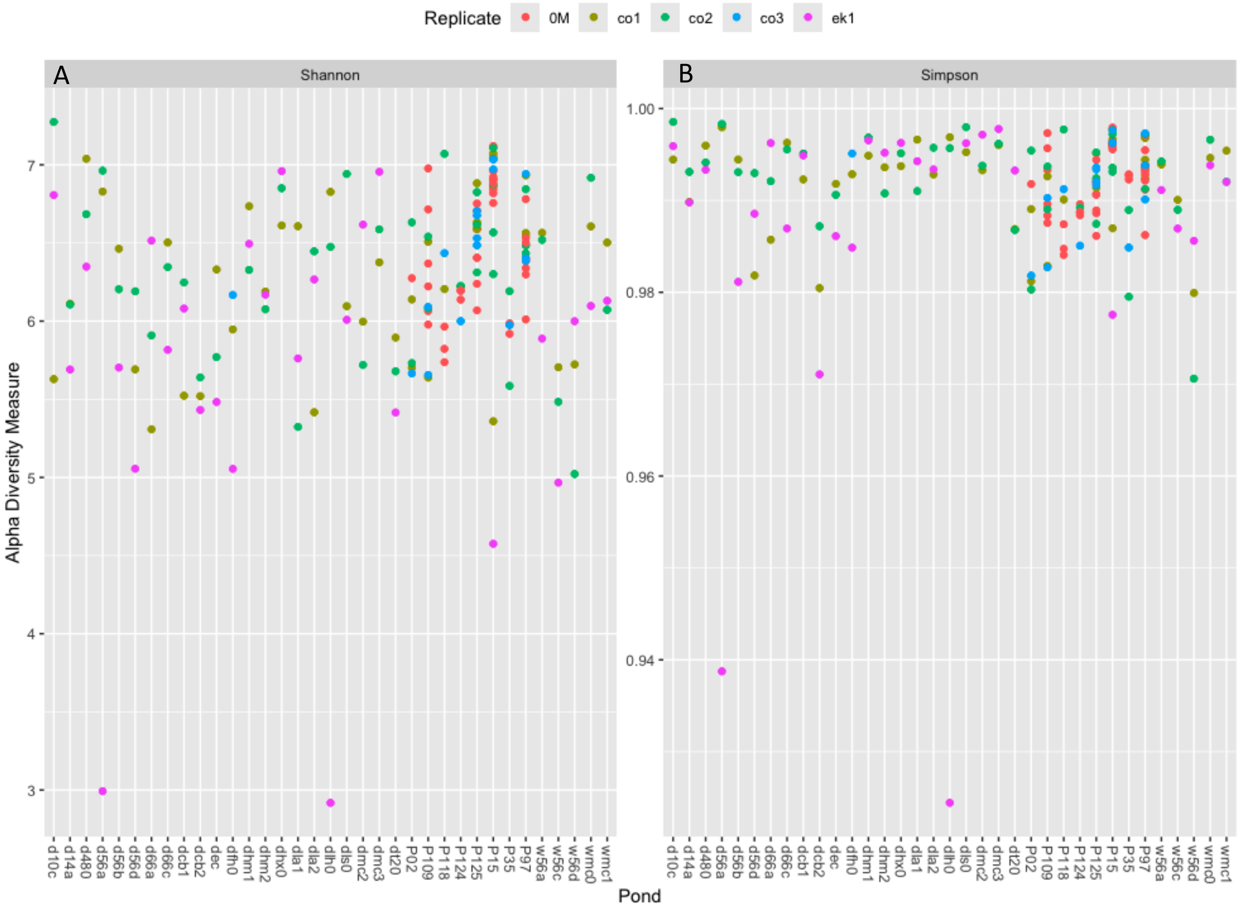

Figure S1: Alpha diversity analysis based on the total number of species derived from 16S rRNA gene-based sequencing of each pond type during the open water season 2021 of 40 ponds. A & B: Species richness and evenness (using Chao1 and Shannon's indices) were calculated using the phyloseq package to find the alpha diversity (n=175). 0m: Soil samples acquired immediately inland from the water's edge; co1, 2, 3: Core samples from ~ 5 cm water depth; ek1: Sediment samples taken from the middle of the ponds using Ekman (Zahir et. al., unpublished data)

Table S2: Sampling depth and Good's Coverage. This table summarizes the number of sequences, singletons, and Good's coverage for each sample. Good's coverage is calculated using 100 * (1 - n_sings / n_seqs), where n_sings is the number of singletons and n_seqs is the total number of sequences (Schloss, 2019). The results reflect the completeness of sequencing and sampling depth across different samples.

Samples	Number of Sequence	Number of Singleton	Goods' Coverage	Samples	Number of Sequence	Number of Singleton	Goods' Coverage
P02A0M052021	84837	0	100.0000	P125COFF052021	69712	1	99.9986
P02AOFF052021	97659	1	99.9990	P125COFF072021	104697	5	99.9952
P02B0M052021	88294	1	99.9989	P125COFF092021	89856	1	99.9989
P02BOFF052021	107523	1	99.9991	P15A0M052021	83532	1	99.9988
P02C0M052021	102552	1	99.9990	P15A0M072021	86722	2	99.9977
P02COFF052021	57608	2	99.9965	P15A0M092021	83464	0	100.0000
P109A0M052021	122531	0	100.0000	P15AOFF052021	95551	2	99.9979
P109A0M072021	84118	1	99.9988	P15AOFF072021	73753	0	100.0000
P109AOFF052021	91300	0	100.0000	P15AOFF092021	97034	0	100.0000
P109AOFF072021	104656	0	100.0000	P15B0M052021	95060	5	99.9947
P109B0M052021	94464	1	99.9989	P15B0M072021	92171	2	99.9978
P109B0M072021	107882	1	99.9991	P15B0M092021	87477	0	100.0000
P109BOFF052021	111236	3	99.9973	P15BOFF052021	118135	3	99.9975
P109BOFF072021	89495	2	99.9978	P15BOFF072021	62287	1	99.9984
P109C0M052021	97277	0	100.0000	P15BOFF092021	73560	2	99.9973
P109C0M072021	96670	0	100.0000	P15C0M052021	101413	5	99.9951
P109COFF052021	99814	2	99.9980	P15C0M072021	105885	2	99.9981
P109COFF072021	84044	1	99.9988	P15C0M092021	95928	2	99.9979
P118A0M052021	106052	1	99.9991	P15COFF052021	91118	1	99.9989
P118AOFF052021	107277	3	99.9972	P15COFF072021	98818	7	99.9929
P118B0M052021	111061	0	100.0000	P15COFF092021	105859	2	99.9981
P118BOFF052021	84840	0	100.0000	P35A0M052021	112767	0	100.0000
P118C0M052021	96410	0	100.0000	P35AOFF052021	98673	2	99.9980
P118COFF052021	94988	1	99.9989	P35B0M052021	106184	2	99.9981
P124A0M052021	93573	2	99.9979	P35BOFF052021	67536	1	99.9985
P124AOFF052021	117843	2	99.9983	P35C0M052021	109067	2	99.9982
P124B0M052021	91360	2	99.9978	P35COFF052021	114730	2	99.9983
P124BOFF052021	122929	6	99.9951	P97A0M052021	96785	1	99.9990
P124C0M052021	102011	4	99.9961	P97A0M072021	95637	1	99.9990
P124COFF052021	95720	6	99.9937	P97A0M092021	102570	2	99.9981
P125A0M052021	116894	5	99.9957	P97AOFF052021	93802	5	99.9947
P125A0M072021	102957	5	99.9951	P97AOFF072021	104741	2	99.9981
P125A0M092021	84381	0	100.0000	P97AOFF092021	83431	3	99.9964
P125AOFF052021	115625	7	99.9939	P97B0M052021	73115	2	99.9973
P125AOFF072021	103818	3	99.9971	P97B0M072021	117153	3	99.9974
P125AOFF092021	90570	3	99.9967	P97B0M092021	96661	1	99.9990
P125B0M052021	112807	2	99.9982	P97BOFF052021	95961	5	99.9948
P125B0M072021	96786	2	99.9979	P97BOFF072021	100601	5	99.9950
P125B0M092021	119876	2	99.9983	P97BOFF092021	79273	5	99.9937
P125BOFF052021	114000	6	99.9947	P97C0M052021	102528	3	99.9971
P125BOFF072021	98123	2	99.9980	P97C0M072021	96111	4	99.9958
P125BOFF092021	84693	1	99.9988	P97C0M092021	100279	3	99.9970
P125C0M052021	86228	0	100.0000	P97COFF052021	98109	3	99.9969
P125C0M072021	75326	4	99.9947	P97COFF072021	90112	2	99.9978
P125C0M092021	114538	3	99.9974	P97COFF092021	100635	3	99.9970

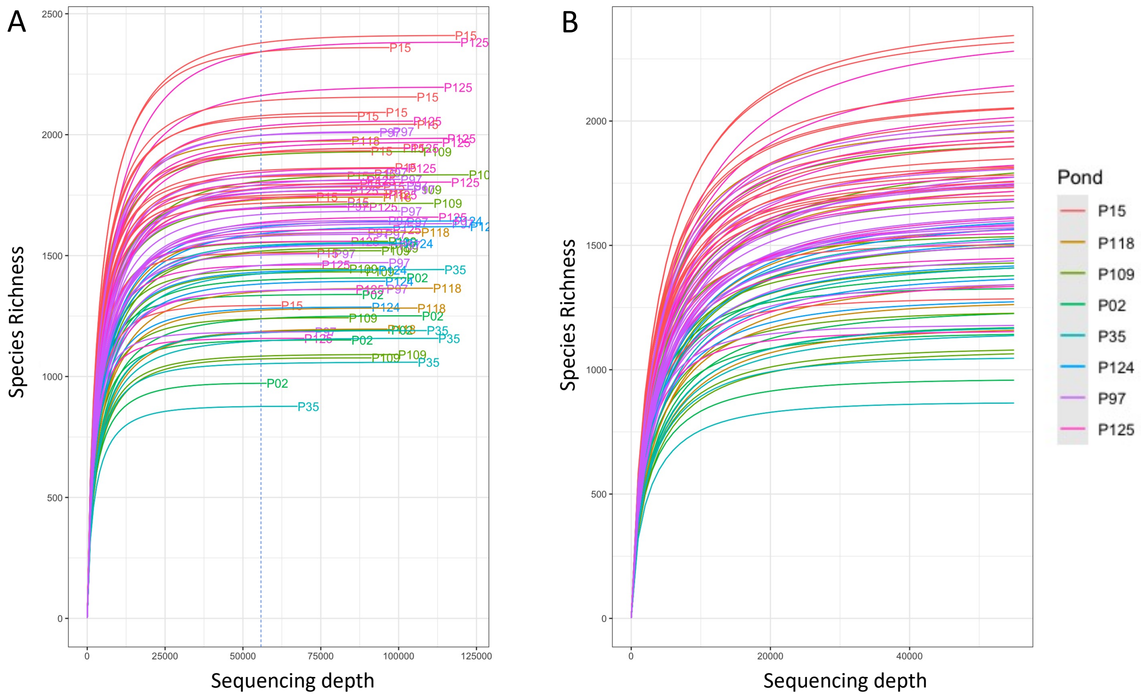

Figure S2: Rarefaction curves before and after sample rarefaction. A: Rarefaction curves generated using the rarecurve() function from the vegan package for all samples. Curves flatten before or at a sequencing depth of 57608 (blue dotted line). B: Rarefying results using the rarefy_even_depth() function from the phyloseq package (v1.44.0; McMurdie and Holmes, 2013) with a sample size of 57,608 (calculated as min(rowSums(otu_table)) with 1,000 iterations and 50 steps). The number of ASVs decreased from 25,274 to 24,389.

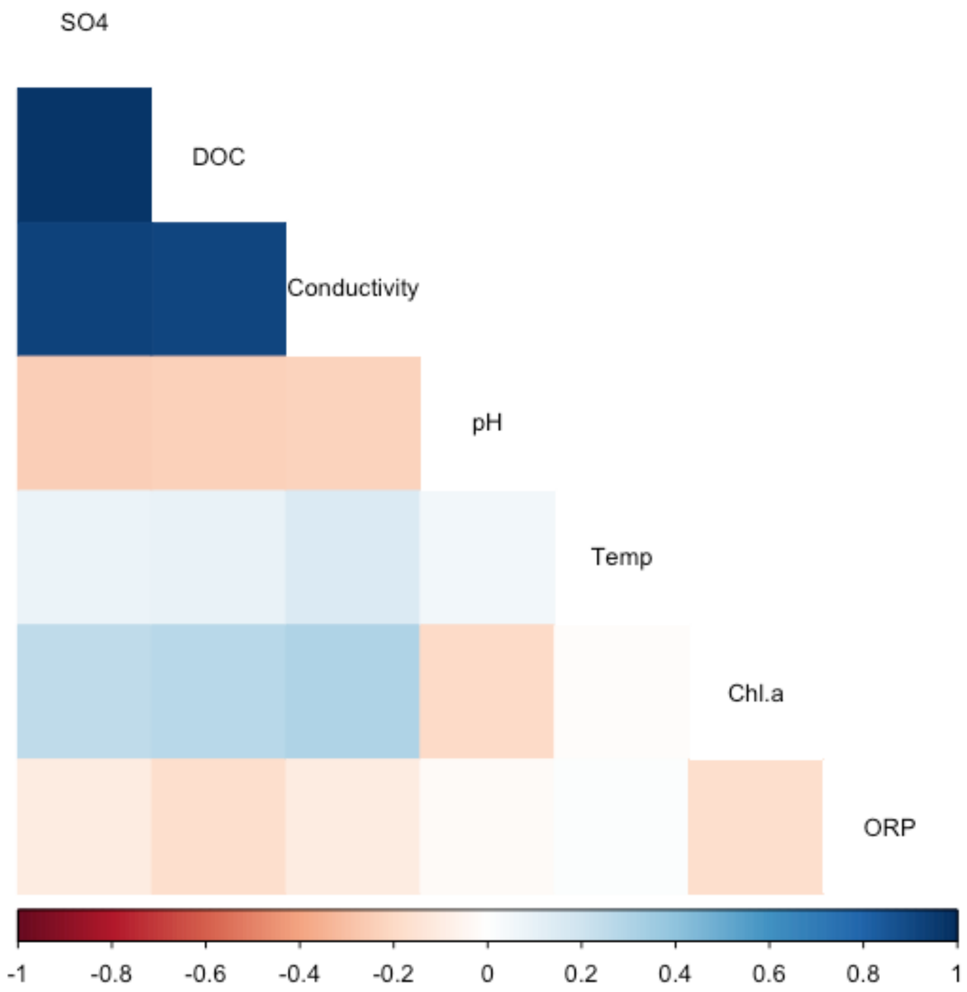

Figure S3: Correlation matrix of geochemistry variables using Spearman's correlation coefficient. Color intensity represents the strength of correlation: darker colors indicate stronger correlations, with positive correlations in blue and negative correlations in red. Spearman's correlation was calculated using the R base package, stats (v4.2.1; R Core Team, 2022).
SO4 = Sulfate concentration (mg/L), DOC = Dissolved organic carbon concentration (mg/L), Conductivity (uS/cm), Temp = Temperature (°C), Chl.a = Chlorophyll a (ug/L), ORP = Oxidation reduction potential (mV).

Table S3: Principal components of geochemistry variables values and eigenvalues across eight ponds during the open water season 2021 (n=48). The significant values > 0.30 are shown in bold. SO_4^{2-} = Sulfate concentration (mg/L), DOC = Dissolved organic carbon concentration (mg/L), Conductivity (uS/cm), Temp = Temperature (°C), Chl.a = Chlorophyll a (ug/L), ORP = Oxidation reduction potential (mV).

Variables	PC1	PC2	PC3	PC4	PC5	PC6	PC7
SO_4^{-2}	**-0.5537**	0.1153	-0.1126	0.1152	-0.0401	0.1423	**0.7951**
DOC	**-0.5578**	0.0585	-0.0342	0.1147	0.0374	**0.6248**	**-0.5283**
Conductivity	**-0.5506**	0.1445	-0.0398	0.0661	-0.1088	**-0.7577**	-0.2895
pH	0.1724	**0.3427**	**0.4204**	**0.7048**	**-0.4215**	0.0401	-0.0006
Temp	-0.0625	**0.4821**	**0.4774**	**-0.6728**	-0.2744	0.0843	0.0226
Chl.a	-0.1383	**-0.7280**	0.1954	-0.1083	**-0.6323**	0.0266	0.0159
ORP	0.1617	0.2869	**-0.7359**	-0.0892	**-0.5764**	0.0749	-0.0628
Explained variance (Eigenvalue) %	42.46	16.49	15.49	13.23	9.95	1.35	0.99
Cumulative %	42.46	58.96	74.45	87.69	97.64	99	100

Table S4: The p-values from t-tests comparing the high and low concentration of SO_4^{2-} and DOC relative to their 25th, 50th, and 75th percentile cut-offs. Percentile cut-offs were calculated using the quantile () function in R, which determines the values below which a given percentage of observations fall. Specifically, the 25th percentile represents the value below which 25% of the data points lie, the 50th percentile (median) is the value below which 50% of the data points lie, and the 75th percentile represents the value below which 75% of the data points lie. T-tests were then conducted to assess significant differences between groups with concentrations above and below each cut-off. The resulting p-values indicate the statistical significance of the differences in concentrations. [SO_4^{2-}] = Sulfate concentration (mg/L), [DOC] = Dissolved organic carbon concentration (mg/L)

Parameter	Cut-off	p-value
[SO_4^{2-}]	25th Percentile (3347.25)	0.0059
[SO_4^{2-}]	50th Percentile (6089.10)	0.0254
[SO_4^{2-}]	75th Percentile (8540.90)	0.1409
[DOC]	25th Percentile (60.85)	0.0050
[DOC]	50th Percentile (114.40)	0.0272
[DOC]	75th Percentile (135.10)	0.0138

TableS5: List of *Euryarchaeota* and *Halobacterota* members from 16S rRNA gene-based sequencing during the open water season of 2021 with their mechanisms of methanogenesis.

Kingdom	Phylum	Class	Order	Family	Genus	Type of methanogens	Mechanism	Study
**Archaea**	**Euryarchaeota**	Methanobacteria	Methanobacteriales	Methanobacteriaceae	Methanobacterium	Hydrogenotrophic	From CO_2 and H_2 to methane	(Li et al., 2017)
**Archaea**	**Halobacterota**	Methanosarcinia	Methanosarciniales	Methanosaetaceae	Methanosaeta	Acetoclastic	Split acetate to methane and CO_2	(Carr et al., 2018; Smith and Ingram-Smith, 2007)
**Archaea**	**Halobacterota**	Methanosarcinia	Methanosarciniales	Methanosarcinaceae	Methanosarcina	Generalist (Hydrogenotrophic, acetoclastic, methylotrophic)	From CO_2 and H_2 to methane; split acetate to methane and CO_2; transferring the methyl group followed by reduction to methane.	(Jetten et al., 1992)
**Archaea**	**Halobacterota**	Methanocellia	Methanocellales	Methanocellaceae	Rice Cluster I	Hydrogenotrophic	From CO_2 and H_2 to methane	(Conrad et al., 2006)
**Archaea**	**Halobacterota**	Methanomicrobia	Methanomicrobiales	Methanoregulaceae	Methanoregula	Hydrogenotrophic	From CO_2 and H_2 to methane	(Bräuer et al., 2011; Treitli et al., 2023)
**Archaea**	**Halobacterota**	Methanocellia	Methanocellales	Methanocellaceae	Methanocella	Hydrogenotrophic	From CO_2 and H_2 to methane	(Sakai et al., 2011)
**Archaea**	**Halobacterota**	Methanosarcinia	Methanosarciniales	Methanoperedenaceae	Candidatus Methanoperedens	Methanotrophic	Metabolize methane	(McIlroy et al., 2023)
**Archaea**	**Halobacterota**	Methanosarcinia	Methanosarciniales	Methanosarcinaceae	Methanolobus	methylotrophic methanogens	Transferring the methyl group followed by reduction to methane	(Tsola et al., 2024)
**Archaea**	**Halobacterota**	Methanosarcinia	Methanosarciniales	Methanosarcinaceae	Methanomethylovorans	methylotrophic methanogens	Transferring the methyl group followed by reduction to methane	(Jiang et al., 2005; Lomans et al., 1999)

**Archaea**	**Halobacterota**	Methanomicrobia	Methanomicrobiales	Methanospirillaceae	Methanospirillum	Hydrogenotrophic	From CO_2 and H_2 to methane	(Parshina et al., 2014)
**Archaea**	**Halobacterota**	Methanomicrobia	Methanomicrobiales	Methanoregulaceae	Methanolinea	Hydrogenotrophic	From CO_2 and H_2 to methane	(Sakai et al., 2012)
**Archaea**	**Euryarchaeota**	Thermococci	Methanofastidiosales	Methanofastidiosaceae	Candidatus Methanofastidiosum	methylotrophic methanogens	Transferring the methyl group followed by reduction to methane	(Nobu et al., 2016)
**Archaea**	**Halobacterota**	Methanomicrobia	Methanomicrobiales	Methanoregulaceae	Methanosphaerula	Hydrogenotrophic	From CO_2 and H_2 to methane	(Cadillo-Quiroz et al., 2015)

Table S6: Comparison between ponds' alpha diversity and phylogenetic diversity values. Chao1 and Shannon's p-value was calculated using ANOVA followed by post-hoc analysis and Ses.PD p-value was calculated using the Wilcox test (*stat* package; R Core Team, 2022).
p adj = p-value after adjustment for the multiple comparisons. Ses.PD = Standardized effect size of phylogenetic diversity (calculated using *picante* package; Kembel et al., 2010). The significant values ≤0.05 are shown in bold.

Pond Comparison	p adj (Chao1)	p adj (Shannon)	p value (Ses.PD)
P118 vs P15	0.06	**0.00**	0.82
P109 vs P15	**0.00**	**0.00**	0.88
P125 vs P15	0.86	**0.00**	**0.01**
P97 vs P15	0.10	**0.00**	0.09
P124 vs P15	**0.02**	**0.00**	0.54
P35 vs P15	**0.00**	**0.00**	0.38
P02 vs P15	**0.00**	**0.00**	0.41
P109 vs P118	1.00	1.00	0.89
P125 vs P118	0.41	0.26	**0.01**
P97 vs P118	0.96	0.33	0.20
P124 vs P118	1.00	1.00	1.00
P35 vs P118	0.40	0.78	0.70
P02 vs P118	0.43	0.76	0.31
P125 vs P109	0.10	0.21	**0.00**
P97 vs P109	0.80	0.30	0.09
P124 vs P109	1.00	1.00	0.82
P35 vs P109	0.28	0.37	0.49
P02 vs P109	0.30	0.35	0.34
P97 vs P125	0.82	1.00	0.58
P124 vs P125	0.24	0.17	**0.01**
P35 vs P125	**0.00**	**0.00**	0.08
P02 vs P125	**0.00**	**0.00**	**0.01**
P124 vs P97	0.86	0.23	0.22
P35 vs P97	**0.01**	**0.00**	0.28
P02 vs P97	**0.01**	**0.00**	0.10
P35 vs P124	0.57	0.86	0.59
P02 vs P124	0.59	0.85	0.13
P02 vs P35	1.00	1.00	0.70

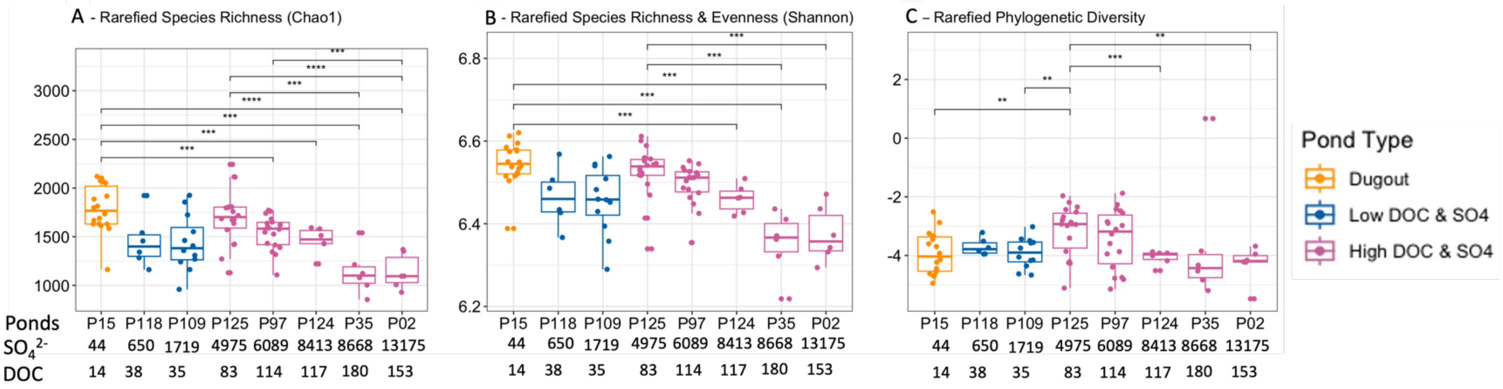

Figure S4: Rarefied versions at the depth of 57608 of alpha and phylogenetic diversity analysis based on the total number of species derived from 16S rRNA gene-based sequencing of each pond type during the open water season 2021. A & B) Species richness and evenness (using Chao1 and Shannon's indices) were calculated using the phyloseq package to find the alpha diversity. C) Phylogenetic diversity (using Ses.PD) was calculated using the picante package to find phylogenetic diversity (n=90). Each box plot shows the smallest (lower box) and largest (upper box) values for the first and third quartiles (25% and 75%), the medians (middle line), the upper and lower whisker (no further than 1.5 inter-quartile range) and points beyond whiskers (outliers). Each color represents a pond type (Low DOC & SO4 in blue, High DOC & SO4 in pink, Dugout in orange). SO_4^{2-} = Mean sulfate concentrations (mg/L), DOC = Mean dissolved organic carbon concentrations (mg/L), Ses.PD = Standardized effect size of phylogenetic diversity. Significance levels: **** $p < 0.0001$, *** $p < 0.001$, ** $p < 0.01$, * $p < 0.05$, calculated by t-test and adjusted using Bonferroni correction. Note: non-significant comparisons are not shown in the figure.

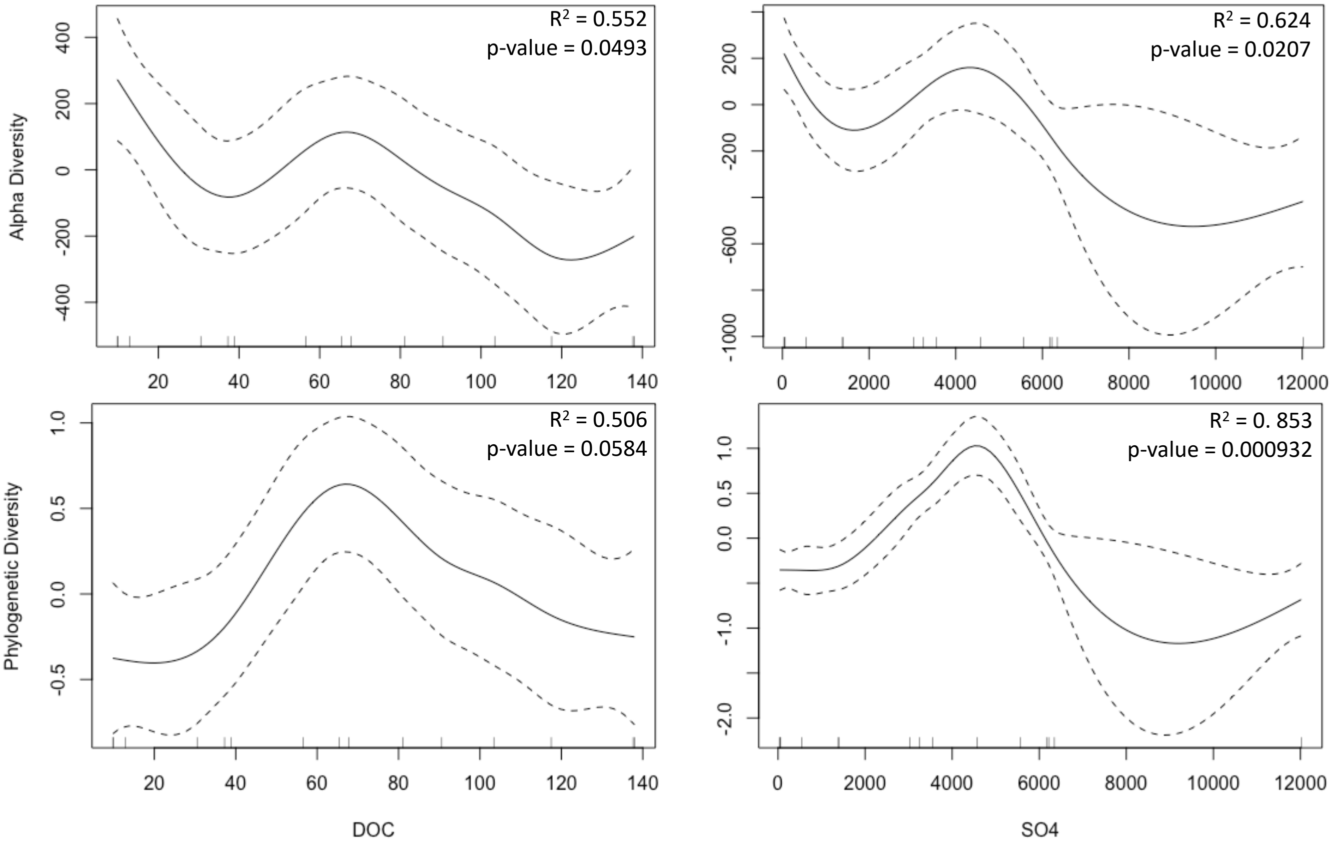

Figure S5: Generalized Additive Models (GAMs) illustrate the correlations of dissolved organic carbon (DOC, left) and sulfate (SO4, right) with alpha diversity (richness based on the Chao1 index, upper panel) and phylogenetic diversity (based on ses.PD, lower panel). Each plot includes the smoothed function of the predictor variable along with its uncertainty (dotted lines). Additionally, the model's goodness-of-fit (R^2 values) indicate the proportion of variance explained, and p-values show the significance of the predictor variables. GAM was calculated using *gam()* function from *mgcv* package (v 1.8-42; Wood, 2023)

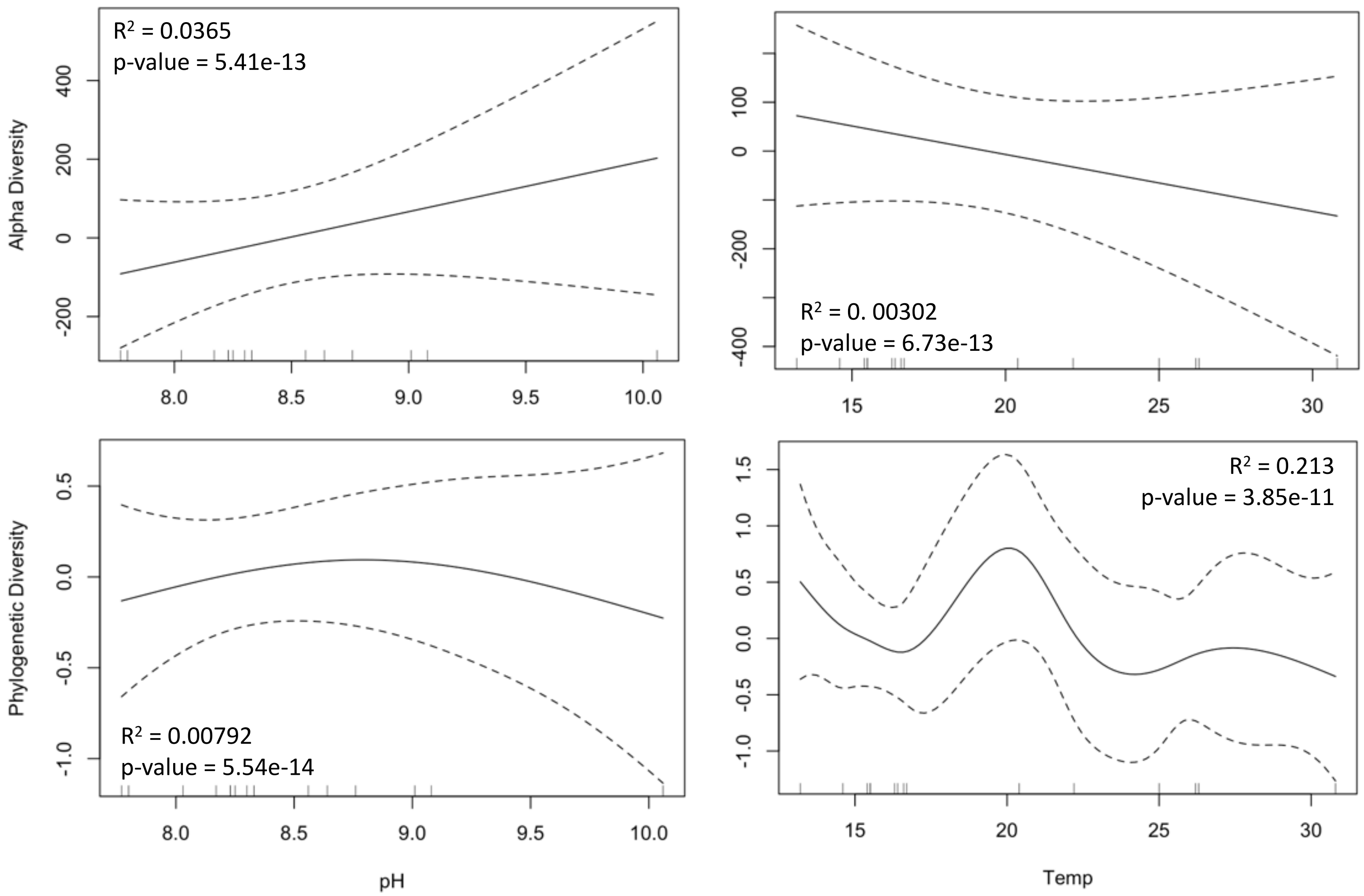

Figure S6: Generalized Additive Models (GAMs) illustrate the correlations of pH (left) and temperature (Temp, right) with alpha diversity (richness based on the Chao1 index, upper panel) and phylogenetic diversity (based on ses.PD, lower panel). Each plot includes the smoothed function of the predictor variable along with its uncertainty (dotted lines). Additionally, the model's goodness-of-fit (R^2 values) indicate the proportion of variance explained, and p-values show the significance of the predictor variables. GAM was calculated using *gam()* function from *mgcv* package (v 1.8-42; Wood, 2023)

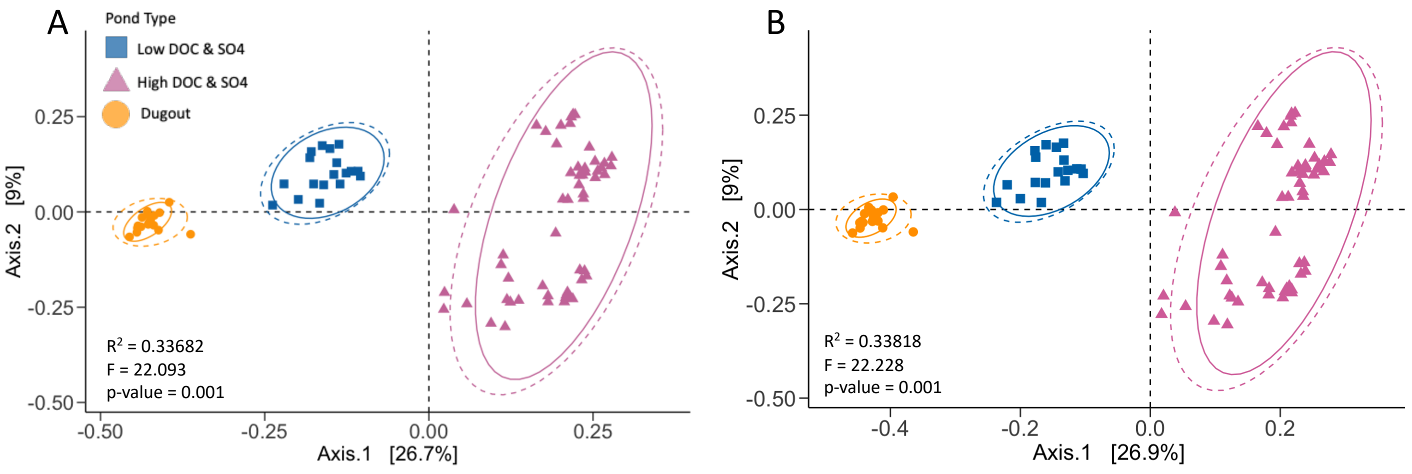

Figure S7: Principal coordinate analysis (PCoA) of 16S rRNA gene-based microbial community composition (n=90) A: Before rarefaction, B: After rarefaction. Points represent individual samples, and each color/shape represents a pond type (Low DOC & SO4 = blue/square, High DOC & SO4 = pink/triangle, Dugout = orange/circle). Ellipses represent 95% confidence intervals around the centroid (solid line = t distribution; dashed line = normal distribution) calculated using the phyloseq package (v1.44.0; McMurdie and Holmes, 2013). The squared correlation coefficient (r^2) and p-value were calculated using PERMANOVA with the adonis2() function from the vegan package (v2.6-4; Oksanen et al., 2015).

References

Bräuer, S.L., Cadillo-Quiroz, H., Ward, R.J., Yavitt, J.B., Zinder, S.H., 2011. Methanoregula boonei gen. nov., sp. nov., an acidiphilic methanogen isolated from an acidic peat bog. International Journal of Systematic and Evolutionary Microbiology 61, 45–52. https://doi.org/10.1099/ijs.0.021782-0

Cadillo-Quiroz, H., Browne, P., Kyrpides, N., Woyke, T., Goodwin, L., Detter, C., Yavitt, J.B., Zinder, S.H., 2015. Complete Genome Sequence of Methanosphaerula palustris E1-9CT, a Hydrogenotrophic Methanogen Isolated from a Minerotrophic Fen Peatland. Genome Announcements 3, 10.1128/genomea.01280-15. https://doi.org/10.1128/genomea.01280-15

Carr, S.A., Schubotz, F., Dunbar, R.B., Mills, C.T., Dias, R., Summons, R.E., Mandernack, K.W., 2018. Acetoclastic Methanosaeta are dominant methanogens in organic-rich Antarctic marine sediments. The ISME Journal 12, 330–342. https://doi.org/10.1038/ismej.2017.150

Conrad, R., Erkel, C., Liesack, W., 2006. Rice Cluster I methanogens, an important group of *Archaea* producing greenhouse gas in soil. Current Opinion in Biotechnology, Environmental biotechnology/Energy biotechnology 17, 262–267. https://doi.org/10.1016/j.copbio.2006.04.002

Jetten, M.S.M., Stams, A.J.M., Zehnder, A.J.B., 1992. Methanogenesis from acetate: a comparison of the acetate metabolism in *Methanothrix soehngenii* and *Methanosarcina* spp. FEMS Microbiology Letters 88, 181–197. https://doi.org/10.1016/0378-1097(92)90802-U

Jiang, B., Parshina, S.N., van Doesburg, W., Lomans, B.P., Stams, A.J.M., 2005. Methanomethylovorans thermophila sp. nov., a thermophilic, methylotrophic methanogen from an anaerobic reactor fed with methanol. Int J Syst Evol Microbiol 55, 2465–2470. https://doi.org/10.1099/ijs.0.63818-0

Kembel, S.W., Cowan, P.D., Helmus, M.R., Cornwell, W.K., Morlon, H., Ackerly, D.D., Blomberg, S.P., Webb, C.O., 2010. Picante: R tools for integrating phylogenies and ecology. Bioinformatics 26, 1463–1464. https://doi.org/10.1093/bioinformatics/btq166

Li, X.-X., Mbadinga, S.M., Liu, J.-F., Zhou, L., Yang, S.-Z., Gu, J.-D., Mu, B.-Z., 2017. Microbiota and their affiliation with physiochemical characteristics of different subsurface petroleum reservoirs. International Biodeterioration & Biodegradation 120, 170–185. https://doi.org/10.1016/j.ibiod.2017.02.005

Lomans, B.P., Maas, R., Luderer, R., Camp, H.J.M.O. den, Pol, A., Drift, C. van der, Vogels, G.D., 1999. Isolation and Characterization of Methanomethylovorans hollandica gen. nov., sp. nov., Isolated from Freshwater Sediment, a Methylotrophic Methanogen Able To Grow on Dimethyl Sulfide and Methanethiol. Applied and Environmental Microbiology 65, 3641. https://doi.org/10.1128/aem.65.8.3641-3650.1999

McIlroy, S.J., Leu, A.O., Zhang, X., Newell, R., Woodcroft, B.J., Yuan, Z., Hu, S., Tyson, G.W., 2023. Anaerobic methanotroph 'Candidatus Methanoperedens nitroreducens' has a pleomorphic life cycle. Nat Microbiol 8, 321–331. https://doi.org/10.1038/s41564-022-01292-9

McMurdie, P.J., Holmes, S., 2013. phyloseq: An R Package for Reproducible Interactive Analysis and Graphics of Microbiome Census Data. PLOS ONE 8, e61217. https://doi.org/10.1371/journal.pone.0061217
Nobu, M.K., Narihiro, T., Kuroda, K., Mei, R., Liu, W.-T., 2016. Chasing the elusive Euryarchaeota class WSA2: genomes reveal a uniquely fastidious methyl-reducing methanogen. The ISME Journal 10, 2478–2487. https://doi.org/10.1038/ismej.2016.33
Oksanen, J., Blanchet, F.G., Kindt, R., Legendre, P., Minchin, P., O'Hara, B., Simpson, G., Solymos, P., Stevens, H., Wagner, H., 2015. Vegan: Community Ecology Package. R Package Version 2.2-1 2, 1–2.
Parshina, S.N., Ermakova, A.V., Bomberg, M., Detkova, E.N., 2014. Methanospirillum stamsii sp. nov., a psychrotolerant, hydrogenotrophic, methanogenic archaeon isolated from an anaerobic expanded granular sludge bed bioreactor operated at low temperature. Int J Syst Evol Microbiol 64, 180–186. https://doi.org/10.1099/ijs.0.056218-0
R Core Team, 2022. R: A Language and Environment for Statistical Computing [WWW Document]. URL https://www.gbif.org/tool/81287/r-a-language-and-environment-for-statistical-computing (accessed 9.30.22).
Sakai, S., Ehara, M., Tseng, I.-C., Yamaguchi, T., Bräuer, S.L., Cadillo-Quiroz, H., Zinder, S.H., Imachi, H., 2012. Methanolinea mesophila sp. nov., a hydrogenotrophic methanogen isolated from rice field soil, and proposal of the archaeal family Methanoregulaceae fam. nov. within the order Methanomicrobiales. International Journal of Systematic and Evolutionary Microbiology 62, 1389–1395. https://doi.org/10.1099/ijs.0.035048-0
Sakai, S., Takaki, Y., Shimamura, S., Sekine, M., Tajima, T., Kosugi, H., Ichikawa, N., Tasumi, E., Hiraki, A.T., Shimizu, A., Kato, Y., Nishiko, R., Mori, K., Fujita, N., Imachi, H., Takai, K., 2011. Genome Sequence of a Mesophilic Hydrogenotrophic Methanogen Methanocella paludicola, the First Cultivated Representative of the Order Methanocellales. PLoS ONE 6, e22898. https://doi.org/10.1371/journal.pone.0022898
Schloss, P.D., 2019. Good's Coverage [WWW Document]. https://mothur.org. URL https://mothur.org/wiki/coverage/
Smith, K.S., Ingram-Smith, C., 2007. Methanosaeta, the forgotten methanogen? Trends in Microbiology 15, 150–155. https://doi.org/10.1016/j.tim.2007.02.002
Treitli, S.C., Hanousková, P., Beneš, V., Brune, A., Čepička, I., Hampl, V., 2023. Hydrogenotrophic methanogenesis is the key process in the obligately syntrophic consortium of the anaerobic ameba Pelomyxa schiedti. ISME J 17, 1884–1894. https://doi.org/10.1038/s41396-023-01499-6
Tsola, S.L., Zhu, Y., Chen, Y., Sanders, I.A., Economou, C.K., Brüchert, V., Eyice, Ö., 2024. Methanolobus use unspecific methyltransferases to produce methane from dimethylsulphide in Baltic Sea sediments. Microbiome 12, 3. https://doi.org/10.1186/s40168-023-01720-w
Wood, S., 2023. mgcv: Mixed GAM Computation Vehicle with Automatic Smoothness Estimation.
